# A chromosome-level genome assembly of *Plantago ovata*

**DOI:** 10.1038/s41598-022-25078-5

**Published:** 2023-01-27

**Authors:** Lina Herliana, Julian G. Schwerdt, Tycho R. Neumann, Anita Severn-Ellis, Jana L. Phan, James M. Cowley, Neil J. Shirley, Matthew R. Tucker, Tina Bianco-Miotto, Jacqueline Batley, Nathan S. Watson-Haigh, Rachel A. Burton

**Affiliations:** 1 School of Agriculture, Food and Wine, University of Adelaide, Waite Campus, Urrbrae, SA Australia; 2 South Australian Genomics Centre (SAGC), Adelaide, SA Australia; 3 Australian Genome Research Facility, Victorian Comprehensive Cancer Centre, Melbourne, VIC 3000 Australia; 4 IP Australia, PO Box 200, Woden, ACT 2606 Australia; 5 School of Biological Sciences, University of Western Australia, Crawley, WA 6009 Australia; 6 Research Center for Genetic Engineering, Research Organization for Life Sciences and Environment, National Research and Innovation Agency (BRIN), Bogor, 16911 Indonesia

**Keywords:** Plant molecular biology, Data mining, Data processing, Genome, Genomics

## Abstract

*Plantago ovata* is cultivated for production of its seed husk (psyllium). When wet, the husk transforms into a mucilage with properties suitable for pharmaceutical industries, utilised in supplements for controlling blood cholesterol levels, and food industries for making gluten-free products. There has been limited success in improving husk quantity and quality through breeding approaches, partly due to the lack of a reference genome. Here we constructed the first chromosome-scale reference assembly of *P. ovata* using a combination of 5.98 million PacBio and 636.5 million Hi-C reads. We also used corrected PacBio reads to estimate genome size and transcripts to generate gene models. The final assembly covers ~ 500 Mb with 99.3% gene set completeness. A total of 97% of the sequences are anchored to four chromosomes with an N50 of ~ 128.87 Mb. The *P. ovata* genome contains 61.90% repeats, where 40.04% are long terminal repeats. We identified 41,820 protein-coding genes, 411 non-coding RNAs, 108 ribosomal RNAs, and 1295 transfer RNAs. This genome will provide a resource for plant breeding programs to, for example, reduce agronomic constraints such as seed shattering, increase psyllium yield and quality, and overcome crop disease susceptibility.

## Introduction

**Figure 1:**
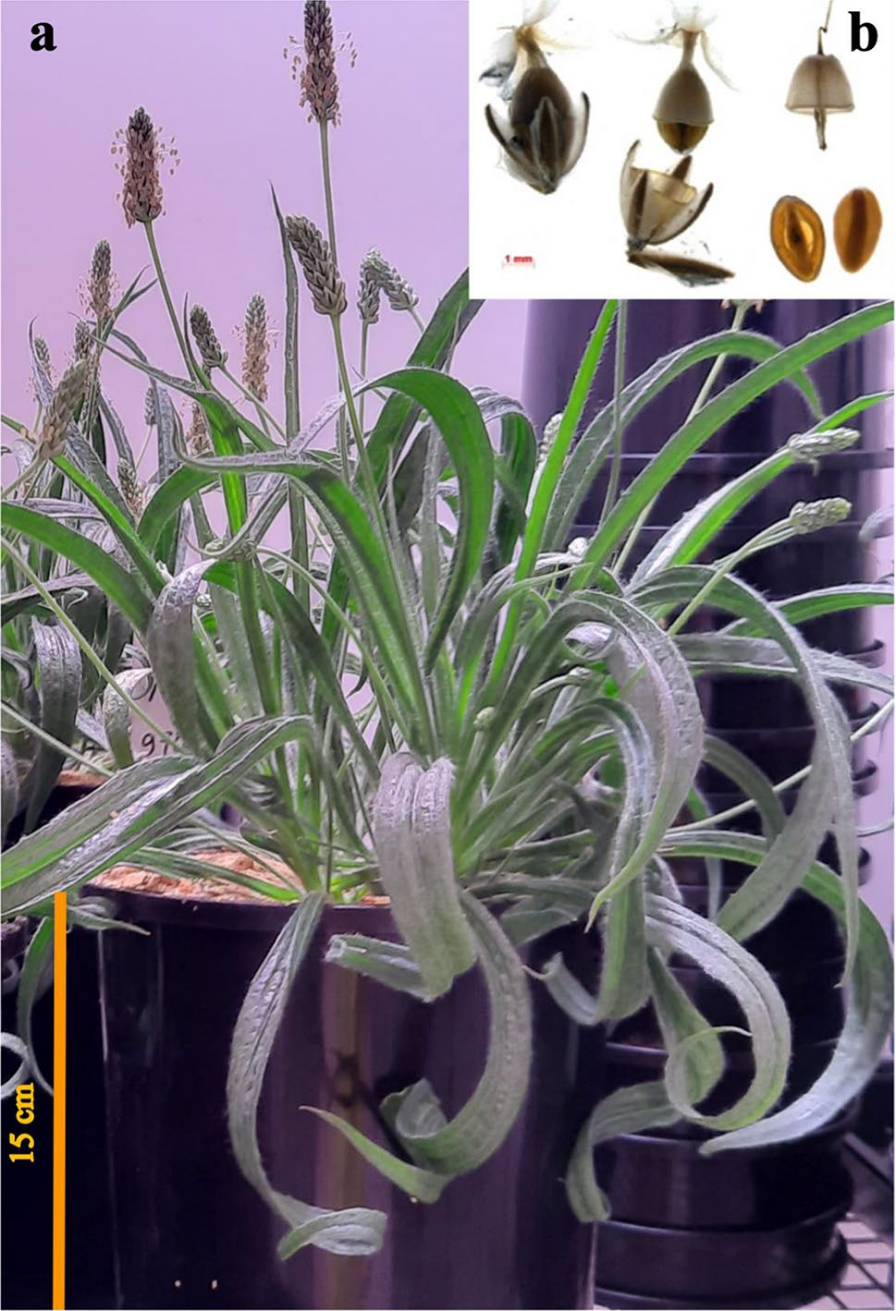
*Plantago ovata*. (**a**) A two-and-a-half-month-old plant. (**b**) Capsules containing two seeds each are fully ripened and shatter easily at around 25 days post anthesis (DPA).

*Plantago ovata* (Fig. 1) seed husk, commonly called psyllium or Isabgol, has a long history of use in human health as dietary fibre when ingested^1,2^ and in food industries as a primary stabiliser in products such as ice cream, and as a gluten substitute in baking^3^. As a commercially valuable plant, many attempts have been made to develop higher-yielding varieties with larger seed size, higher husk content, non-shattering capsules, synchronous maturity, and resistance to abiotic (e.g., drought and frost) and biotic stresses (e.g., downy mildew)^4,5^. As the primary producer and exporter, India initiated a *P. ovata* breeding program as early as 1976 in the Pilwai tract of North Gujarat, while trials to establish best agronomic practices were undertaken in Australia in 1985 in the Ord River Irrigation Area (ORIA), Kununurra region, Western Australia^6^. However, many studies reported that conventional breeding approaches had not significantly improved seed or psyllium production^7–9^. Genetic improvement of this plant is challenging because *P. ovata* has a narrow genetic base, a small number of chromosomes (2n = 8) enriched in heterochromatin, low chiasmata frequency, low recombination index and a high selfing rate^8–13^. As a result, this plant is sensitive to environmental changes that may threaten the supply chain and increase the global price of psyllium.

Exposure to gamma irradiation has been reported to successfully induce phenotypic variation in *P. ovata*^13–15^. *P. ovata* var. ‘Mayuri’ is one example of a gamma-irradiated mutant with valuable traits, including early maturation with pigment markers guiding the right timing for harvesting, combined with high seed and husk production^14^. However, before this cultivar was patented in 2003, the evaluation period was very long, requiring three generations for selfing (M1-M3), three generations for vegetative propagation (M4-M6) and two years for pilot-scale trials^14^. This period could be significantly reduced if the candidate genes related to the favourable traits were known. One way to identify candidate genes is to use RNA sequencing to generate transcriptomic data. Since 2010, at least six studies have deposited *P. ovata* RNA-seq raw data in the Sequence Read Archive (SRA) at the National Center for Biotechnology (NCBI) (Supplementary File 1: Table S1). All the studies used de novo transcript assembly because no genome reference was available. Only the *P. ovata* chloroplast genome has been assembled to date^16^. This helps resolve taxonomic relationships among species but has limited application for genetic improvement. The challenge of using transcriptome assemblies is distinguishing between sequence artefacts and the genes themselves due to alternative splicing producing splice variants. In addition, there is a need to create a transcriptome assembly for every different project as transcripts are tissue and time specific. To provide a universal resource, a reference genome is required.

Here we report the process of generating and utilising a *P. ovata* chromosome level assembly. Continuous long read (CLR) data from Pacific Biosciences (PacBio) was used to create a contig assembly, while a Hi-C approach capturing chromosome conformation was used to guide the scaffolding. We gathered all publically available RNA-seq data and combined it with data generated at the University of Adelaide to predict the gene models. The construction of a *P. ovata* reference genome will help genetic improvement programs for *P. ovata* as well as supporting laboratory-based experiments to better understand the seed biology of this species.

## Results and discussion

### Genome assembly and chromosome identification

A *Plantago ovata* genome reference was generated by utilizing a total of 5.98 M (7 cells, 40.21 Gb, N50 = 10.45 Kb, 50 bp–121.17 Kb) PacBio long reads and 636.5 million (47.74 Gb) Hi-C short-reads. PacBio reads were used to assemble contigs, while Hi-C reads were used to achieve chromosome-level assembly. The final assembly has 876 sequences (500.94 Mb, N50 = 128.87 Mb) (Table 1, Supplementary File 1: Table S2). The four superscaffolds account for 97.29% (487.38 Mb) of the total genome length and the unplaced scaffolds account for only 2.71% (13.55 Mb). Based on the lengths of the scaffolds, we assigned HiC_scaffold_1 (137.73 Mb) as chromosome 1, HiC_scaffold_2 (128.87 Mb) as chromosome 2, HiC_scaffold_3 (114.44 Mb) as chromosome 3, and HiC_scaffold_4 (106.35 Mb) as chromosome 4 (Supplementary File 2).

**Table 1:** Summary of *P. ovata* genome assembly and annotation.

Total assembly size (Mb)	500.94
Total contig number	4301
Contig N50 length (Kb)	249.86
Total scaffolds number	876
Scaffold N50 (Mb)	128.87
Total chromosome number	4
Total chromosome length (Mb)	487.38
GC content (%)	38.40
Gene number (all)	41,820
Gene number (AED > 0.5)	23,638
Repeat content (%)	61.90
LTR Assembly Index (LAI)	10.27
BUSCO assembly	C:99.3% [S:94.1%, D:5.2%], F:0.5%, M:0.2%, n:425*
BUSCO protein-coding genes (AED > 0.5)	C:80.7% [S:78.1%, D:2.6%], F:8.9%, M:10.4%, n:425*

AED, Annotation Edit Distance; LTR, Long Terminal Repeat; C, Complete; S, Complete and single-copy; D, Complete and duplicated; F, Fragmented; M, Missing BUSCOs;*, Viridiplantae_odb10, BUSCO v5.4.3.

**Figure 2:**
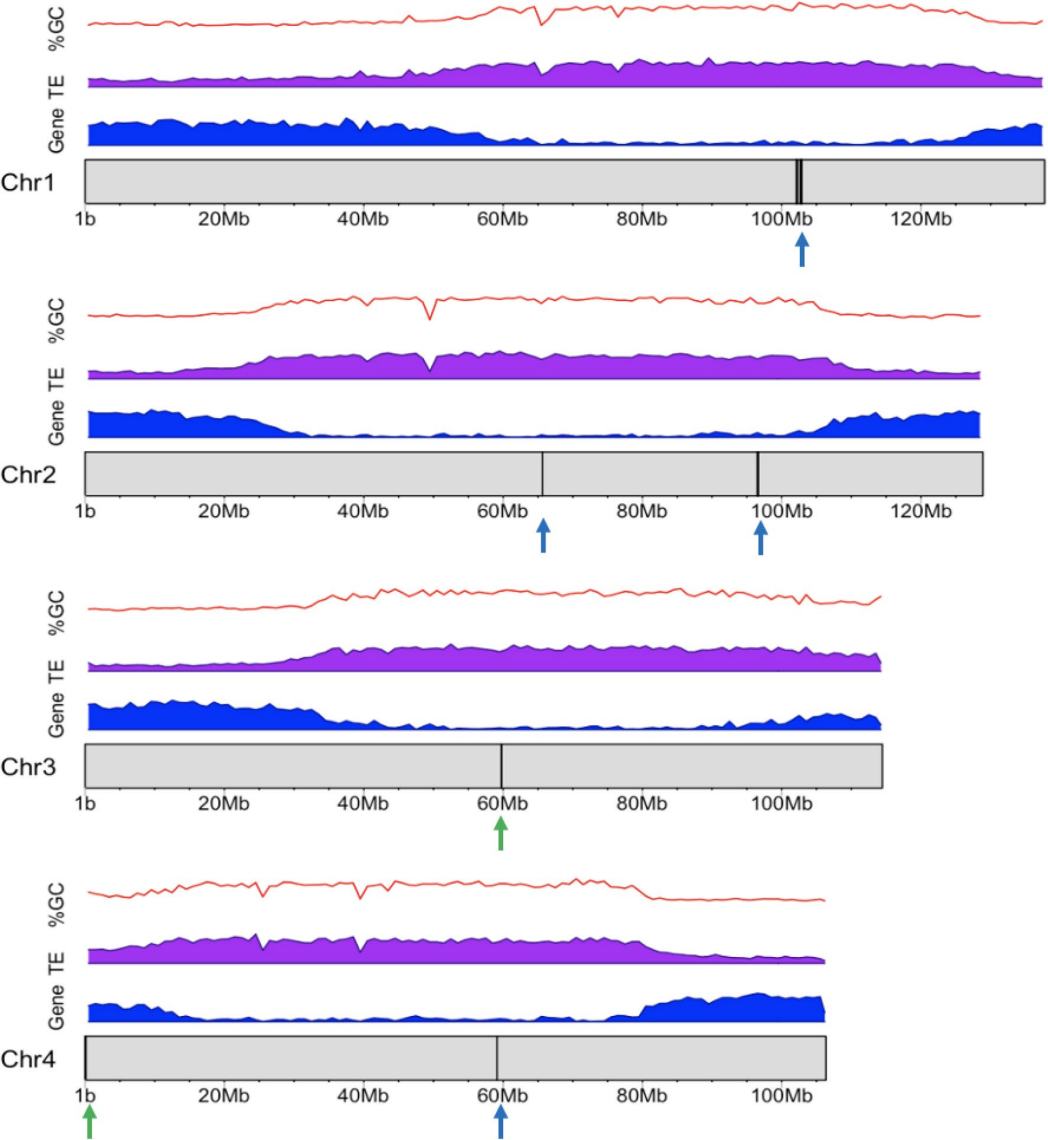
Gene density (blue), TE (Class I and II) density (purple), % GC (red), distribution of 5S (blue arrows) and 45S (green arrows) rRNA in the *P. ovata* genome. The figure was generated in R using the karyoploteR library^19^. The x-axis represents genome position (Mb) and the y-axis represents gene density using a sliding window of one megabase in length.

We are confident in labelling HiC_scaffold_1 as chromosome 1 because of the presence of the 5S rDNA cluster^11,17^ and HiC_scaffold_2 as chromosome 2 as it does not contain any 45S rDNA sequences. Only chromosomes 3 and 4 have 45S rDNA sequences (Fig. 2)^11,17^. However, the location of 45S rDNA on chromosome 3 in our assembly, near the middle of the chromosome, is not the same position as that proposed based on ribosomal physical mapping^11^. Previous researchers found 45S rDNA signals at the ends of the short arms of chromosomes 3 and 4. This difference could represent intraspecific variation or missed joins in the assembly. Better quality raw long reads could address the problem of misjoined contigs. In addition, optical mapping technology could be used to validate the orientation of the de novo assembly in the future^18^.

According to the centromere positions, *P. ovata* chromosome 1 is classified as metacentric, chromosome 2 as submetacentric while chromosomes 3 and 4 are subtelocentric^11^. However, in this assembly, the position of the centromeres is not accurately fixed but rather is indicated using euchromatin and heterochromatin patterns. Euchromatin is active chromatin in the genome where more genes are transcribed, while heterochromatin is a less active and highly condensed region on the chromosome (Fig. 2). Dhar et al.^12^ reported that euchromatic areas are located at the distal ends of all chromosomes and cover one arm of chromosome 1 entirely. Our results agree with this but also provide additional information (Fig. 2), defining heterochromatic regions from 60 to 125 Mb on chromosome 1, from 30–105 Mb on chromosome 2, 40–100 Mb on chromosome 3 and 15–80 Mb on chromosome 4. These heterochromatic regions contain a high density of class I and II transposable elements (TE) (Fig. 2). The statistics for repeat content (61.90%, Supplementary File 1: Table S3) and proportion of total gene lengths (32.06%) that account for less than one-third of chromosome lengths (Supplementary File 1: Table S4) support the earlier finding using C binding and fluorescence in situ hybridization (FISH) methods indicating that most of the regions in the *P. ovata* genome are heterochromatin containing highly repetitive DNA^12^.

### Genome size

To predict the *P. ovata* genome size, corrected PacBio reads were used. The result from *k*-mer analysis (21-mer) shows that the estimated haploid genome size is 551.02 Mb using findGSE v0.1.0^20^ while genomescope2 v2.0^21^ predicted 415.78 Mb (Supplementary File 3). Our assembly size (500.94 Mb) (Table 1) sits within the range of estimated haploid genome size using the *k*-mer method. The *P. ovata* genome size has been previously estimated using flow cytometry and reported in three different studies. Badr et al.^22^ reported diploid *P. ovata* from Cairo has a genome of between 484.11 Mb (C value: 0.495 pg) and 523.23 Mb (C value: 0.535 pg). Pramanik and Raychaudhuri^10^ studied an Indian cultivar (Anand) and reported a size of 537.9 Mb (C value: 0.55 pg), whilst Dhar et al.^12^ estimated the *P. ovata* genome size at about 621 Mb (C value: 0.635 pg). Potentially the range in sizes could be due to use of different methods^12^ but could also represent intraspecific variation. Schmuths et al.^23^ found significant differences covering a 1.1-fold range between the genome size of 21 Arabidopsis accessions.

### Genome quality

The *P. ovata* genome assembly presented here is high quality as defined by several parameters. Comparison between the final assembly and the corrected PacBio reads using the KAT comp tool (kat v2.4.1)^24^ showed that the assembly contains mostly single copy numbers of the reads (Supplementary File 4). Despite having 876 scaffolds, four scaffolds with chromosome lengths accounting for 97.29% of the total haploid genome size were detected and visualised using a Hi-C interaction heatmap (Supplementary File 2), indicating that the assembly is highly contiguous. The shortest scaffold length at 50% of the total genome length (N50) is 128.87 Mb which is chromosome 2 (Table 1, Supplementary File 2). The scaffold N50 value is far higher than the average length of a *P. ovata* gene at 3,840 bp (Supplementary File 1: Table S5) indicating a much higher chance of generating complete gene models. This is supported by a BUSCO assembly completeness value (BUSCO v5.4.3) of 99.3%, where only one out of 425 genes present in a Viridiplantae cohort (viridiplantae_odb10, creation date: 2020–09-10) is missing in this assembly (Table 1). The percentage of publically available genomic short-read Illumina data (SRR10076762) mapped to our genome assembly is 95.81%, while the portion of our genomic long-read PacBio data (SRR14643405) mapped back to the assembly is 92.25%. In addition, the high mapping rate of reads from RNA-seq data to this assembly (up to 96.10%) will facilitate accurate data interpretation by preventing false positives in downstream analyses such as for transcriptomics (Supplementary File 1: Table S6)^25^.

We used the LAI score to evaluate the continuity of our assembly where the program requires at least 0.1% intact LTR-RTs and 5% LTR-RTs as a proportion of the total genome size^26^. Ou et al.^26^ evaluated 103 genomes with contents of intact LTR-RTs ranging from 0.28% to 18.34% and total amounts of LTR-RTs from 5.49 to 69.38%. Our assembly meets these criteria with 8.38% intact and 52% total LTR-RTs. This assembly has an LAI score of 10.27 (Supplementary File 5). Based on the classification of assembled repeat sequences using the LAI score^26^, our assembly can be classified as a reference (10 $\le$ LAI $\le$ 20). Advances in technology to produce longer reads with higher accuracy could further improve the current assembly to gold or even platinum standard.

Of note is that this assembly has a lower LAI score (10.27) than the raw LAI (15.90) (Supplementary File 5). About 25% (26/103) of genomes studied^26^ show the same trend. All these genomes, including our assembly (96.34%), have a whole genome LTR identity higher than 94%. It has been suggested that those species with recent LTR-RT amplifications provide more intact raw LTR elements that are thus represented by a higher LTR identity.

### Mitochondrial DNA insertions

Three regions in this assembly were detected as originating from mitochondrial sequences based on contamination screening during genome submission to the NCBI database. Two regions are in HiC_scaffold_1 (chromosome 1), with one in HiC_scaffold_2 (chromosome 2). The lengths are 250 bp, 149 bp, and 177 bp (Supplementary File 6). However, PacBio long reads span these three regions with no breaks suggesting that they are genuinely part of the nuclear genome (Supplementary File 6). Michalovova et al.^27^ similarly reported insertions of nuclear mitochondrial DNA (NUMT) and nuclear plastid DNA (NUPT) in six plant species. They reported the insertions were localised near centromeres in rice and Arabidopsis. During manual curation of the *P. ovata* annotation file, genes from the chloroplast and mitochondria were found in the nuclear assembly, suggesting these three regions are most likely to be NUMT. Further research is needed to investigate gene transfer from organelles to the nuclear genome to characterise NUMT and NUPT in *P. ovata*.

### Repeat content estimation and identification

The *P. ovata* genome appears to contain 61.90% (310.10 Mb) repeats with long terminal repeat (LTR) retrotransposons comprising the highest proportion (200.59 Mb, 40.04%) (Supplementary File 1: Table S3). Two out of three major groups of LTR retrotransposons were detected in the assembled genome. They are Ty1/Copia (98.64 Mb, 19.69%) and Gypsy (101.64 Mb, 20.29%). There are 366 sequences defined as satellites (98,9 Kb, 0.02%). Less than 1% (3.49 Mb) of the repeat content is simple repeats. Simple repeats TTTAGGG identified as a typical plant telomere sequence^11^, were located at the end of all chromosomes, while AAACCCT, the canonical or reverse complement of the telomere repeat, was found at the beginning of the chromosomes. Other telomeric variants were also found in this assembly, such as TTTGGGG, TTTCGGG, TTCAGGG, TTTTAGGG and AACCCGG (Supplementary File 7).

### GC content

The guanine (G) and cytosine (C) content of DNA has been reported to play an important role in gene regulation and can be associated with how organisms adapt to their environment^28,29^. Šmarda et al.^28^ observed that plants with GC-rich DNA were more adaptative in extreme climates. Overall, the GC content of this genome is about 38.4% (Table 1, Supplementary File 8). Comparison between GC content, gene density and TE class I and II in 1 Mb-wide sliding windows showed that the average GC content was higher by 3% (40%) in the area with high TE density compared to regions with high gene density (37%) (Fig. 2, Supplementary File 1: Table S7).

Dhar et al.^9^ originally stated that the *P. ovata* genome had 55% GC content, adjusting this four years later to an AT content of 59.7%^12^ and dropping the GC content to 40.3%. The latest study^12^ was conducted using flow cytometry (FCM). Šmarda et al.^28,30^ compared the GC content of 11 rice species using FCM versus sequence data. They found that GC contents from sequence data are consistently lower than those from the flow cytometer. The different methods could explain why the GC content reported by Dhar et al.^12^ is slightly higher than our calculation of 38.4%, based on genomic sequences. However, Dhar et al.^12^ and this study agree that the *P. ovata* genome is AT-rich. As AT base pairs have lower thermal stability than the GC base pairs^28^, having low GC content could signify that the plant is potentially less adaptive to extreme climates. Wang et al.^31^ found that plant domestication contributed to higher A and T content in maize and soybean compared to their wild relatives. Commercial *P. ovata* accessions could display the same increase in AT content due to domestication but breeding efforts have not been as intense in this species as for other crops. To test this hypothesis, we could measure and compare the GC content of Australian native *Plantago* species described in Cowley et al.^32^ to the commercial accessions of *P. ovata*.

The GC content of the CDS, at 44.3%, (Supplementary File 9) is higher by 6% compared to genomic GC content (Table 1, Supplementary File 8). Kotwal et al.^33^ also found that the GC content of the *P. ovata* transcripts in their study was higher than the genomic GC content. However, as they only extracted and sequenced one tissue type (ovaries) this may not be a valid comparison. Kotwal et al.^33^ also compared the GC content of *P. ovata* transcripts with *A. thaliana*, rice, tomato, and *Eucalyptus*. They classified *P. ovata* and *Eucalyptus* (dicot/eudicot) in the same group as rice (monocot) with GC contents of 45–50% while *A. thaliana* and tomato (eudicot) had a lower GC content ranging from 40 to 45%^33^. However, *P. ovata* has a unimodal distribution (one peak) (Fig. 3 in Kotwal et al.^33^, Supplementary File 1: Table S8). In contrast, rice has a bimodal distribution (two peaks) (Fig. 3 in Kotwal et al.^33^) so they should not be classified in the same group. Singh et al.^29^ studied the GC content from 20 plant genomes and ranked the highest GC content as coming from grass genomes (including rice), followed by a non-grass monocot and then finally from eudicots. Their results also showed that the eudicot genome has a unimodal distribution while grass monocots have a bimodal distribution^29^. Bimodal distribution is shaped by highly heterogenous GC content among genes in the grass genomes, giving one peak with GC-rich genes and another with GC-poor genes^29^. In contrast, eudicots show low variability or homogenous GC content among genes resulting in only one peak^29^. High GC content has been found to be positively correlated with high recombination sites^34^, which may be important for breeding strategies.

**Figure 3:**
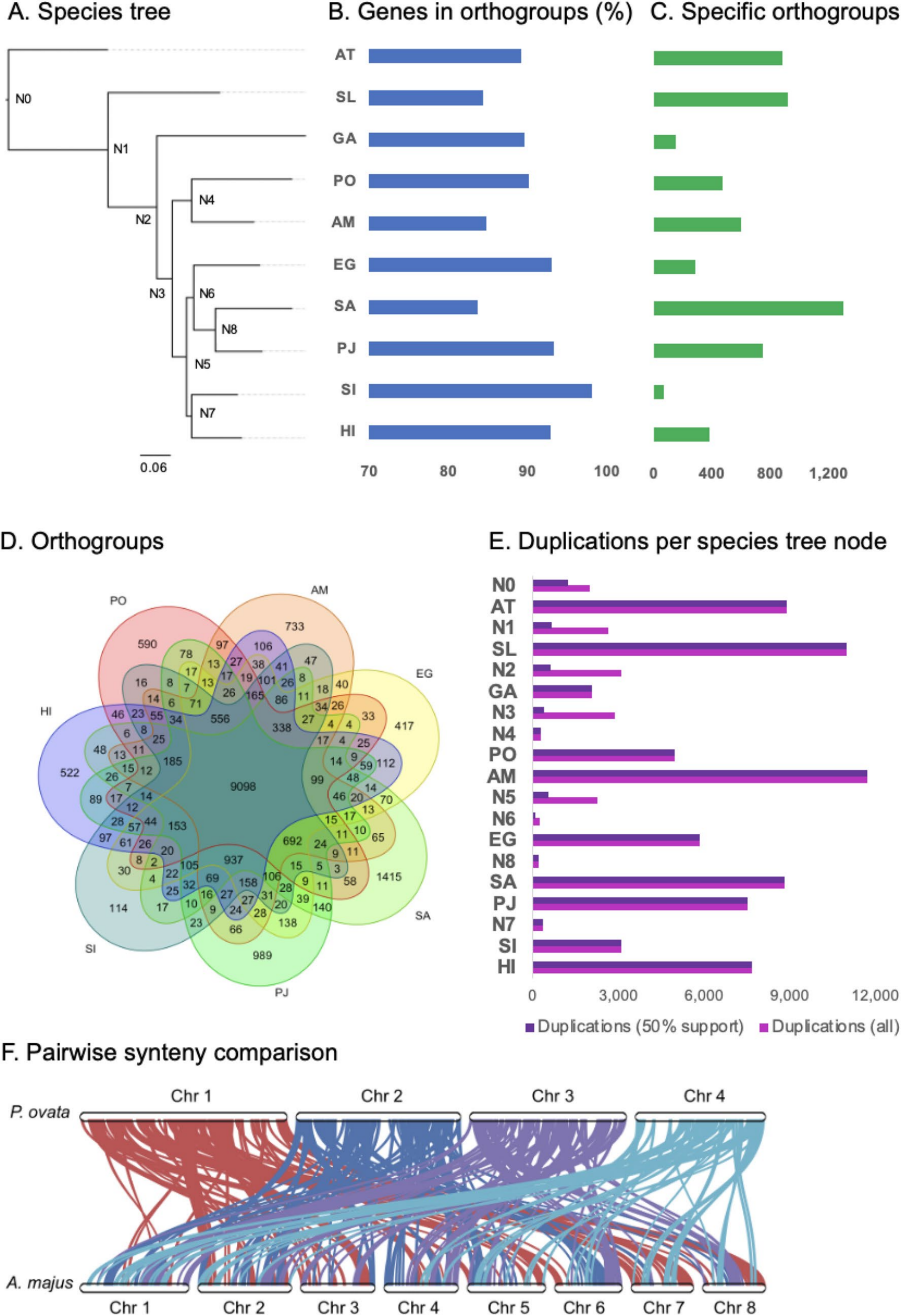
Comparative genomic analyses between *P. ovata* (PO) with other Laminales species (GA, *Genlisea aurea*; AM, *Antirrhinum majus*; EG, *Erythranthe guttata*; SA, *Striga asiatica*; PJ, *Phtheirospermum japonicum*; HI, *Handroanthus impetiginosus*; SI, *Sesamum indicum*), one Brassicales (AT, *Arabidopsis thaliana*) and one Solanales (SL, *Solanum lycopersicum*). (**A**) Bar charts for each species in B & C were aligned to the corresponding species in the species tree. Bootstrap values of the species tree of each node are one except N3 is 0.76. (**B**) Percentage of genes from each species assigned to orthogroups. (**C**) The number of species-specific orthogroups. (**D**) Venn diagrams of orthogroups from 7 species (GA, PO, AM, EG, SI, and AT). (**E**) The number of gene duplication events per internal and terminal nodes from the species-based-phylogenetic tree. (**F**) Pairwise synteny comparison between *P. ovata* and *A. majus*.

### Comparative genomic analysis

The *P. ovata* genome is estimated to contain 41,820 protein-coding genes (Table 1) based on a set of mRNA transcripts from this organism (Supplementary File 1: Table S1 & S6), protein homology sequences from related organisms under Viridiplantae, and ab initio gene prediction using MAKER v2.31.11^35^. However, only 56% (23,638/41,820) of protein-coding genes have an Annotation Edit Distance (AED) less than 0.5. AED values range from 0, with perfect agreement of the annotation to aligned evidence, and 1, with no supporting evidence for the annotation. There is still much room to improve this annotation in the future. However, use of BUSCO v5.4.3^36^ indicates that the completeness of protein-coding genes is still 80.7% (Table 1).

The protein sequence from the longest transcript variant from each of 23,638 genes was then compared with nine other species using OrthoFinder v2.5.4^37^ (Fig. 3). The species tree in Fig. 3A shows that *A. thaliana* (Brassicales) and *Solanum lycopersicum* (Solanales) were the outgroups of the species in Laminales (*Genlisea aurea*, *Plantago ovata*, *Antirrhinum majus*, *Erythranthe guttata*, *Striga asiatica*, *Phtheirospermum japonicum*, *Handroanthus impetiginosus*, and *Sesamum indicium)*. *P. ovata* is closely related to *A. majus* as they belong to the same family, Plantaginaceae.

OrthoFinder v2.5.4^37^ assigned 255,025 genes out of 285,170 (89.4%) from 10 species to 22,916 orthogroups (Supplementary File 1: Table S9). There were 7,281 orthogroups with all ten species present, and 1,003 of these consisted entirely of single-copy genes. The mean orthogroup size is ten genes (Supplementary File 1: Table S9). The percentage of genes from each species assigned to orthogroups (Fig. 3B) ranged from 83.7% to 98.1%, with *P. ovata* at 90.1% (Supplementary File 1: Table S10). There were 5,824 species-specific orthogroups, ranging from 68 orthogroups belonging to *S. indicum* to 1,307 orthogroups of *S. asiatica*. *P. ovata* has 475 specific orthogroups (Supplementary File 1: Table S10). These numbers slightly increased by looking only at all descendant species from branch N3 (Fig. 3D). For example, core orthogroups among these seven species were 9,098, with 590 *P. ovata*-specific orthogroups. *P. ovata* shared the most specific orthogroups with *A. majus* at 97 (Fig. 3D, Supplementary File 1: Table S11), with 41 single-copy genes from these two species. In comparison, twenty-three orthogroups consist of one single *P. ovata* gene but more than one *A. majus* gene (Supplementary File 1: Table S11). The most extreme is *P. ovata* GeneID *Pov_00010246*, which has 115 orthologs in *A. majus*. The number of gene duplication events in *A. majus* is the highest among all species studied and more than that of *P. ovata* gene duplication events (11,735/4962 genes, Fig. 3E). The genome sizes of *P. ovata* (500.94 Mb) and *A. majus* (510 Mb)^38^ are comparable. However, *A. majus* has eight chromosomes^38^, double that of *P. ovata* (4). Using MCscan (jcvi v1.2.11)^39^, 314 syntenic blocks between *P. ovata* and *A. majus* were detected. These blocks are distributed across all *P. ovata* chromosomes: 94 on Chr 1, 81 on Chr 2, 80 on Chr 3, and 59 on Chr 4. Almost all of the four *P. ovata* chromosomes have syntenic regions to the eight *A. majus* chromosomes except there are no blocks on *P. ovata* Chr 4 syntenic to *A. majus* Chr 3 (Fig. 3F). Overall, about 30% of the total *P. ovata* genome does not correlate to syntenic regions in *A. majus* (Supplementary File 10). Single *P. ovata* syntenic blocks that contain only one *A. majus* gene account for 50% of the genome, while 18% of the *P. ovata* genome has two blocks that correlate to a single *A. majus* gene. Conversely, a region containing a single *P. ovata* gene corresponds to one *A. majus* block across 37% of the genome, regions containing two to 43%, and three to 3% of the *A. majus* genome (Supplementary File 10).

### Glycosyltransferase family 61 (GT61) family

Upon hydration, *P. ovata* seeds release mucilage with physicochemical properties that are determined by polysaccharide composition and molecular structure, particularly backbone substitution levels and patterning. *P. ovata* is rich in complex heteroxylan^17,40,41^, composed of a backbone of xylose residues decorated with a variety of side chains typically comprised of arabinose (Ara), xylose (Xyl), and traces of other sugars^40,42^. We used this current genome assembly to identify candidate genes of the glycosyltransferase family 61 (GT61) family, which appear to encode key enzymes involved in arabinose and xylose substitution^43,44^ with a significant impact on final mucilage quantity and quality. Eighteen *PoGT61* sequences from public data were added to the comparative genomic analysis to search for GT61 orthogroups and orthologues.

**Figure 4:**
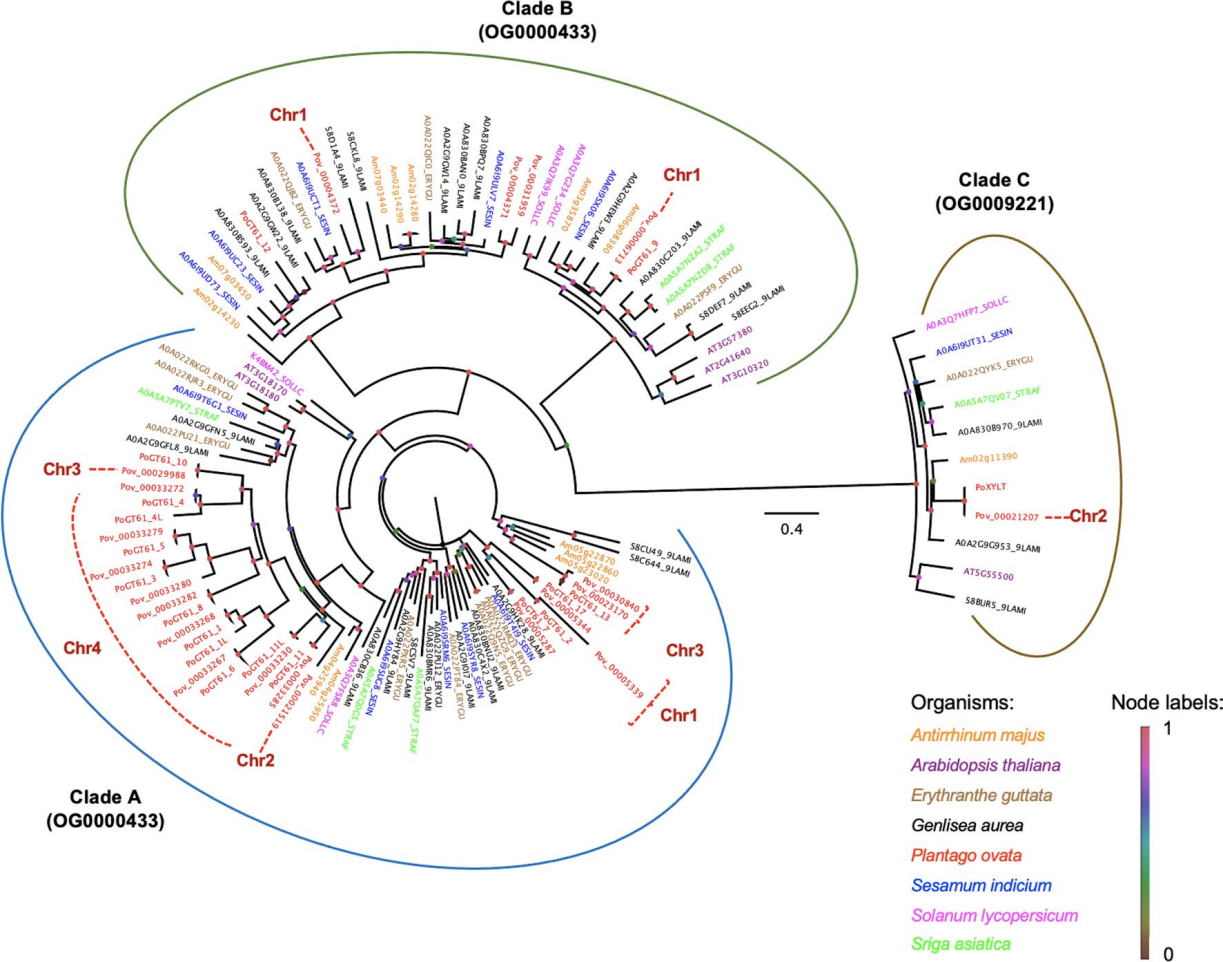
A phylogenetic tree of GT61 protein sequences from selected species was visualised using FigTree v1.4.4. Clades A—C were labelled as per Anders et al.^44^ and Voiniciuc et al.^46^.

Public *P. ovata* GT61 (*PoGT61*) sequences^43,45^ were grouped into three orthogroups, OG0000114, OG0000433, and OG0009221 (Fig. 4, Supplementary File 1: Table S12). Clades A—C were labelled as per Anders et al.^44^ and Voiniciuc et al.^46^. These orthogroups or clades consist of sequences from 8 species out of 10 studied, where OG0009221 (Clade C) has one gene copy for each species, including *PoXYLT* (Fig. 4). Fifteen of the *PoGT61* sequences were grouped into Clade A while only two sequences (*PoGT61_9* and *PoGT61_12*) belonged to Clade B. *PoGT61_1* and *PoGT61_1L* both mapped to *Pov_00033268* whilst *PoGT61_4* and *PoGT61_4L* mapped to *Pov_00033272* respectively. In contrast, *PoGT61_11* and *PoGT61_11L* mapped to different genes, *Pov_00033285* and *Pov_00033230*, respectively. On the other hand, *PoGT61_13* (Clade A) and *PoGT61_12* (Clade B) have sequence similarities to more than one gene in our assembly (Supplementary File 1: Table S12). Thus, the previous analysis of the *P. ovata* contigs derived from the de novo transcriptome assembly^43^ was insufficient to fully resolve the single gene origin of alternative splice variants, but this has now been possible using this reference genome.

In total, there are 19 GT61 genes identified. Nine were clustered on Chr4, five on Chr1, three on Chr3, and two on Chr2. The nine genes located on chromosome 4 are clustered in the phylogenetic tree. These genes were predicted to be xylan arabinosyltransferases (α-1,3-arabinosyltransferase) from the annotation file (Supplementary File 1: Table S12). Heterologous expression of these genes in other species could confirm their function. For example, the heterologous expression of rice and wheat GT61 genes in Arabidopsis increased arabinose substitution and provided gain-of-function evidence for arabinosyltransferase activity^44^. The significantly higher number of *Plantago* GT61 gene duplications has previously been suggested to be linked to the high density/complexity of backbone substitutions on the heteroxylan of *P. ovata* mucilage^43^. Different GT61 enzymes may add specific types of heteroxylan backbone decorations, and the heterologous expression of multiple *Plantago* GT61 genes in tandem, in a suitable host, may reveal such roles.

### Non-coding RNA annotations

We identified 108 ribosomal RNAs (rRNAs), 1,295 transfer RNAs (tRNAs), and 411 non-coding RNAs (ncRNAs). The identified non-coding RNAs (ncRNAs) comprise 328 long non-coding RNAs (lncRNAs), 17 primary transcripts of microRNAs (miRNAs), 48 small nuclear RNAs (snRNAs), 12 small nucleolar RNAs (snoRNAs), 2 ribonuclease mitochondrial RNA processing (RNase MRP) RNAs, and 4 signal recognition particle (SRP) RNAs. Several types of cytoplasmic rRNA are annotated in the genome belonging to 5S, 18S, and 25S classes. The 5S sequences are clustered on chromosome 1 (63 sequences) with only six 5S sequences on chromosome 2, one on chromosome 4 and none on chromosome 3. Ribosomal 45S RNAs are found only on chromosomes 3 and 4 (Fig. 3).

In total, there are 328 lncRNAs in the *P. ovata* genome. They are distributed across four chromosomes with 97 transcripts on chromosome 1, 76 transcripts on chromosome 2, 86 transcripts on chromosome 3, 56 transcripts on chromosome 4, and 13 transcripts on unplaced sequences. Based on the locations of lncRNAs and the nearest mRNAs, we found 320 lncRNA/mRNA pairs in the assembly (Supplementary File 1: Table S13). They can be grouped into four categories, which are 50 antisense genic, 85 antisense intergenic, 88 sense intergenic, and 97 sense genic.

#### Miscellaneous annotations

Overall, all parameters assessed indicate that we have generated a high quality assembled and annotated genome. The genome can be used as a reference, but we also provide Supplementary files that can benefit future research. Supplementary File 1: Table S13 contains information about lncRNA and mRNA candidates for future functional analysis to study how gene expression may be controlled by epigenetic mechanisms. Supplementary File 11 lists annotation for LTR Copia and Gypsy retrotransposons that may be helpful to study *Plantago* domestication. Identified location and sequences of genes linked to histone modifications and DNA methylation can be found in Supplementary File 1: Table S14, providing an additional epigenetic resource. The telomere sequences in Supplementary File 7 can be used for evolutionary analysis as suggested in the review by Peska and Garcia^47^.

## Conclusions

This study has generated the first *P. ovata* genome assembly together with gene annotations. We achieved a chromosome-level assembly using de novo assembly of PacBio CLR data and contig scaffolding utilising Hi-C data. Our assembly is about 500 Mb in size and comprises four chromosomes. This resource will help accelerate *Plantago* breeding programs. Markers can be developed and candidate genes identified related to key phenotypes using Genome-Wide Selection (GWS) or RNA-seq strategies by comparing two distinct genotypes occurring in nature or generated by mutation. Specific regions in the genome can be targeted to improve the quantity and quality of psyllium using the latest technology, such as CRISPR/Cas9 or to select favourable traits in breeding programs.

## Materials and methods

### DNA extraction, library preparation and sequencing

*P. ovata* seeds were obtained from Accolent Dried Herbs, Queensland, Australia^43^. Plants were grown in the glasshouse as per Phan et al.^43^. Leaf tissues from mature plants were used for genomic DNA extraction for PacBio and Hi-C library construction. The study complies with local and national guidelines.

For PacBio sequencing, DNA extraction was achieved by combining protocols from Sikorskaite et al.^48^ and QIAGEN® Genomic-tip Protocols. First, leaf tissues were washed with deionized water and blotted dry before freezing in liquid nitrogen. The tissues were ground into a fine powder using a pre-chilled mortar and pestle. The ground tissues (1 g) were resuspended in 25 mL cold Nuclei Isolation Buffer (NIB) and mixed until completely homogenized (15–30 min). The composition of NIB was as per Sikorskaite et al.^48^. The mixture was filtered through pre-wetted miracloth and left on ice for 20 min. The chlorophyll layer was separated by centrifugation at 18,000 rpm for 20 min at 4 °C. This layer was discarded, and only the pellet was kept. The pellet was resuspended in 25 mL NIB. The remaining chlorophyll layer was separated again by centrifugation. The pellet was resuspended in 2 mL lysis buffer (QIAGEN® Genomic-tip) before adding 4 µL DNAse-free RNase and incubating for 30 min at 37 °C. Proteinase K (0.8 mg/mL) was added to this mixed solution before incubating for one hour at 50 °C with gentle agitation. To remove insoluble debris, the solution was centrifuged for 30 min at 4,000 rpm. The supernatant was treated following QIAGEN® Genomic-tip Protocols. The genomic DNA in TE buffer (pH 7.6) was sent to the Australian Genome Research Facility Ltd (AGRF) for library preparation and PacBio Sequel I (PacBio Sequel System, RRID:SCR_017989).

Hi-C libraries were prepared using the Proximo Hi-C (Plant) Prep Kit (Phase Genomics, Seattle, WA, US). A *P. ovata* plant was incubated in the dark for 48 h before the collection of leaf material. Young leaves (0.2 g) were collected and chopped finely and immediately added to 10 mL of crosslinking solution to crosslink the chromatin. After 15 min of incubation, 100 µL of quenching solution was added, and the samples were incubated again for 20 min while rotating. The leaf material was pelleted by centrifugation, washed with 1 × CRB provided and patted dry before grinding into a fine powder. The ground leaf sample was suspended in cell lysis buffer to release the chromatin, followed by fragmentation of the chromatin, proximity ligation and library preparation which were carried out according to the Proximo Hi-C (Plant) Prep Kit protocol v.2. The final library concentration was determined using a Qubit fluorometer (Invitrogen, Carlsbad, CA, US), while the library quality and size was assessed with the LabChip GX Touch 24 using the HT DNA HiSens Dual Protocol Reagents (PerkinElmer, Hopkinton, MA, US). The final library had a median size of 570 bp. The libraries were sequenced on a HiSeq 2500 System (Illumina, San Diego, CA, US) by GENEWIZ (Suzhou, China) in paired-end mode, generating 150 bp reads (PE 150).

### De novo genome assembly

**Figure 5:**
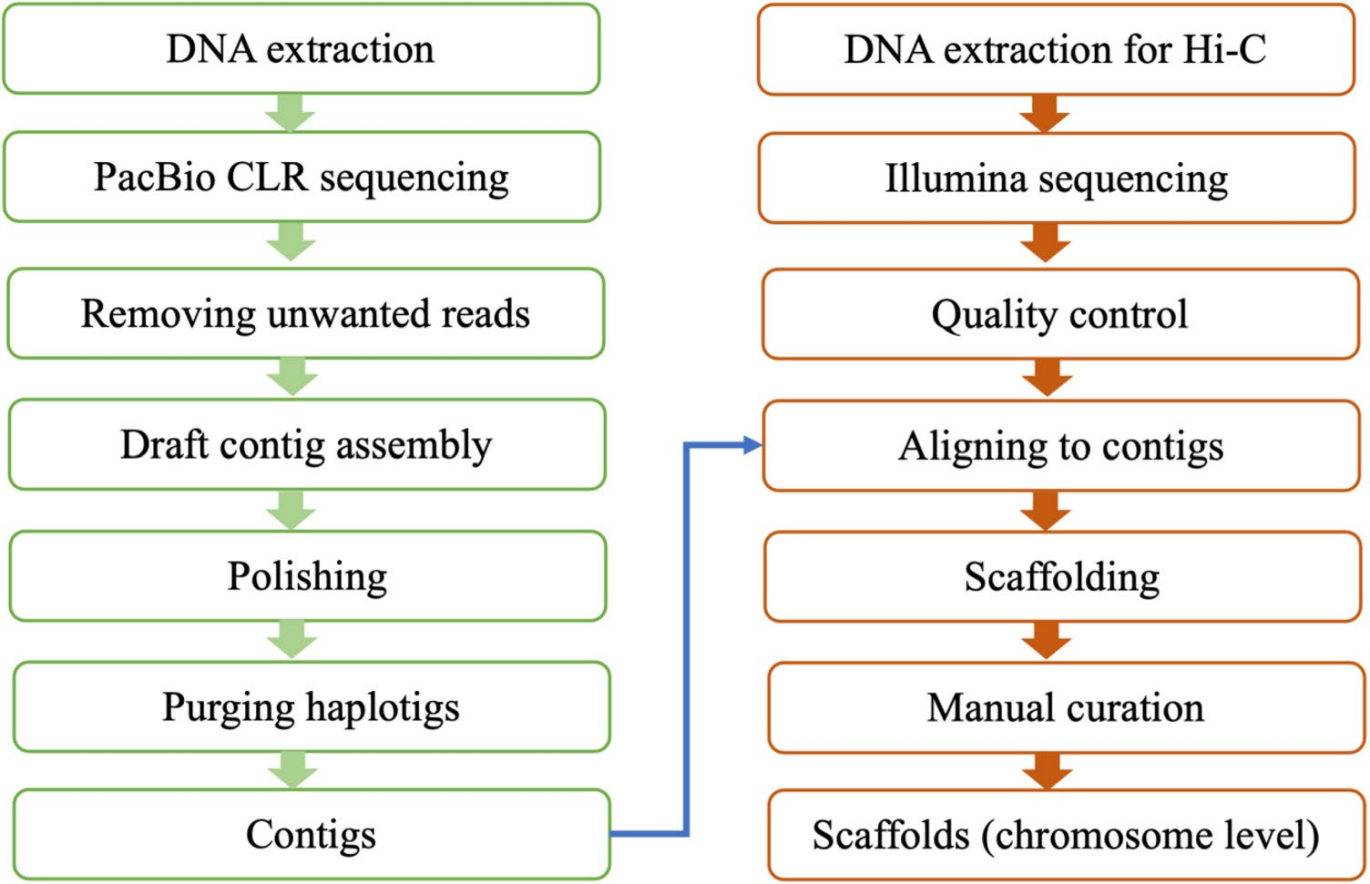
Illustration of the genome assembly strategy.

Continuous long reads (CLRs) from the PacBio platform (~ 76X coverage) were used to assemble the *P. ovata* genome. Several steps were used to process raw reads, including removing unwanted reads, contig assembly, polishing, and purging haplotigs (alternative contigs) (Fig. 5). Firstly, seven unaligned subread BAM files from the PacBio Sequel I System were converted into FASTQ files using bam2fastx v1.3.0 (PacBio Sequel System, RRID:SCR_017989). Unwanted reads (mitochondria and chloroplasts) were removed by filtering out reads that mapped to either the chloroplast genome (GenBank: MH205737.1)^16^ or the sole *P. ovata* mitochondrial gene available at the time (GenBank: EU069524.1). The read alignment was performed using Minimap2 v2.17 (Minimap2, RRID:SCR_018550) with “-ax map-pb” parameters and SAMtools v1.9 (SAMTOOLS, RRID:SCR_002105) used to extract unmapped reads. The total number of sequences and sequence lengths were checked before and after removing unwanted reads using FastQC v0.11.9 (FastQC, RRID:SCR_014583). Reads after cleaning were assembled following a pipeline by Canu v2.1 (Canu, RRID:SCR_015880)^49^ with optimised parameters: “corMhapSensitivity = high corMinCoverage = 0” to correct as many read as possible for coverage > 50X and " corOutCoverage = 200 batOptions = -dg 3 -db 3 -dr 1 -ca 500 -cp 50" to avoid collapsing the genome.

The draft contig assembly was polished with PacBio CLR reads. The subset of CLR reads used for polishing excluded the reads previously identified as being derived from the mitochondria or chloroplasts. Following the mapping of reads to the draft assembly, the polishing step was parallelised to decrease memory requirements and improve wall-time. This was achieved through a scatter–gather approach where each contig was independently processed. Filtered reads were mapped to the draft contig assembly using pbbam v1.6.0 before the polishing steps using pbgcpp v1.0.0 (PacBio Sequel System, RRID:SCR_017989). Circular and bubble contigs were removed from the polished contig assembly using seqtk v1.3 (Seqtk, RRID:SCR_018927).

After polishing, a purge was performed to remove haplotigs. First, the polished contig assembly was indexed, then the clean reads were mapped onto the improved assembly using Minimap2 v2.17 (Minimap2, RRID:SCR_018550) and sorted using SAMtools v1.9 (SAMTOOLS, RRID:SCR_002105). After mapping, purge_haplotigs v1.1.1 (Purge_haplotigs, RRID:SCR_017616) was used to detect and separate the primary and alternative contigs. To improve handling of repetitive regions, a list of contigs that were predicted as repeats from the Canu v2.1 report (Canu, RRID:SCR_015880)^49^ were parsed into the purge_haplotigs pipeline. Cut-offs were applied at “-l 5 -m 70 -h 190”. The clipping option in purge_haplotigs was also used, to find and trim overlapping contigs that may prevent scaffolding.

### Chromosome level assembly

Hi-C data (636.5 million reads, 47.74 Gb) was used to link contigs into a chromosome level assembly. First, the quality of the Hi-C reads was checked using FastQC v0.11.9 (FastQC, RRID:SCR_014583) and Trimmomatic v0.39 (Trimmomatic, RRID:SCR_011848) was used to remove primer sequences. To assess library preparation quality, the pipeline suggested by Phase Genomics (https://phasegenomics.github.io/2019/09/19/hic-alignment-and-qc.html) was followed. The contig assembly was indexed and clean Hi-C reads were aligned to the assembly using bwa v0.7.17 (BWA, RRID:SCR_010910). Reads derived from PCR duplicates were subsequently identified and flagged using samblaster v0.1.26 (SAMBLASTER, RRID:SCR_000468). Read alignments where the read is unmapped, the mate is unmapped, is not a primary alignment, or is a supplementary alignment (SAMtools parameter “-F 2316”) were discarded. The mapped reads were also filtered using matlock v20181227 (https://github.com/phasegenomics/matlock) with default parameters and the QC of the reads were checked before and after filtering (Supplementary File 12). Although both QC reports showed that the Hi-C library was good quality, the filtering increased the numbers of high quality read pairs from 38.68% to 100%. Following the mapping of Hi-C reads, two different tools (SALSA2^50^ and 3D-DNA^51^) were tested for scaffolding performance. Only 3D-DNA yielded chromosome-scale superscaffolds. Firstly, aligning Hi-C clean reads to contig assembly was done using Juicer (Juicer, RRID:SCR_017226). After running the 3D-DNA pipeline, candidate assembly was visualised and reviewed using Juicebox Assembly Tools (JBAT)^51^. Then, a new assembly was generated by running a 3D-DNA post review pipeline. To meet NCBI submission requirements, we removed sequences with less than 200 nucleotides (nt) and reduced the unknown gap length (NNN) from 500 to 100 nt. Chromosomes were numbered from 1 to 4 from longest to shortest.

### Genome size prediction and assembly quality

Corrected PacBio reads generated from Canu v2.1.1 (Canu, RRID:SCR_015880)^49^ were used to predict *P. ovata* genome size using genomescope2 v2.0 (GenomeScope, RRID:SCR_017014)^21^ and findGSE v0.10^20^. The quality of our assembly was assessed using the following parameters: assembly contiguity, gene set completeness, mapping rates of genomic and transcriptomic reads, and assembly continuity. The genome size and N50 value of each assembly stage were calculated using Perl script “n50.pl”^52^. Assembly completeness was measured by Benchmarking Universal Single-Copy Orthologs v5.4.3 (BUSCO, RRID:SCR_015008)^36^ against a Viridiplantae database (viridiplantae_odb10, creation date: 2020–09-10). A test was performed to see if the reads from publically available Illumina genomic data under SRA number SRR10076762 generated by the CSIR-Central Salt and Marine Chemicals Research Institute, India from genotype GI-20 could be mapped to our genome assembly by running Minimap2 v2.17 (Minimap2, RRID:SCR_018550), then sorting and counting mapped reads using SAMtools v1.9 (SAMTOOLS, RRID:SCR_002105). The assembly continuity was also evaluated by calculating the Long Terminal Repeat Assembly Index (LAI) using LTR_retriever^26^.

### Repeat content estimation and identification

Repeats were identified using RepeatModeler v2.6.1 (RepeatModeler, RRID:SCR_015027) and calculated by RepeatMasker v4.1.1 (RepeatMasker, RRID:SCR_012954). Firstly, a custom repeat library (CRL) was built by running RepeatModeler on the *P. ovata* genome against the Dfam transposable element family database (Dfam_3.2). Repeats derived from protein-coding regions were removed from the library. Viridiplantae protein-encoding sequences were obtained from the UniProt Knowledgebase (UniProtKb) database (https://www.uniprot.org/taxonomy/33090, access date 13 Augustus 2021). The transposable element homolog sequences were detected by transposonPSI.pl (http://transposonpsi.sourceforge.net/) and removed by gaas_fasta_removeSeqFromIDlist.pl^53^ from the collected proteome. The filtered proteome was segregated from the repeat library by searching the homologies using BLASTX (BLASTX, RRID:SCR_001653) and excluding them via ProtExcluder.pl^54^. The filtered CRL was then used to calculate the *P. ovata* genome by RepeatMasker following the tutorial by Dainat (https://www.biostars.org/p/411101/#411101). In the final annotated genome, remaining repeat annotations overlapping with protein-coding genes were removed manually based on NCBI’s discrepancy report.

### De novo transcriptome assembly and identification of protein coding genes

*P. ovata* transcripts were generated from a set of RNA-seq data obtained from a range of tissues (88 fastq files) (Supplementary File 1: Table S2). The data was grouped depending on how the library was prepared (Supplementary File 1: Table S2). The first category is paired stranded libraries with Illumina sequencing (56 files). The second contains paired un-stranded libraries with Illumina sequencing (2 files). The third group is a single-stranded library with Illumina (8 files). The fourth is a single un-stranded library with Illumina (12 files) while the fifth group is a single un-stranded library with Roche 454 sequencing (10 files).

The RNA-seq data was filtered by quality checking, trimming, cleaning, and aligning reads to the reference genome to generate accurate gene models. Quality checking was performed using FastQC v0.11.9 (FastQC, RRID:SCR_014583) and MultiQC v1.8 (MultiQC, RRID:SCR_014982) with default parameters. Trimmomatic v0.39 (Trimmomatic, RRID:SCR_011848) was used to remove adapter and PCR primer fragments. Two read groups were treated in the paired-end mode and the other three groups with single-end mode. To remove contaminants, BBDuk, BBmap v38.87 (BBmap, RRID:SCR_016965) was used. Rates of contamination (1.5–94.5%) and mapping (54.9–96.1%) varied across samples (Supplementary File 1: Table S2). Contamination rates were higher for leaf, bract, stem, and capsule tissues (> 20%) than for integument and ovaries (< 20%). To generate a high-quality genome annotation, only RNA-seq data with a high mapping rate of greater than 85% was used, and to be consistent two samples with contamination rates of more than 95% were removed leaving 46 samples (71 fastq files). Clean reads were aligned to the reference genome using STAR v2.7.6a (STAR, RRID:SCR_015899). After mapping reads to the reference genome, transcripts were generated separately for each group using Cufflinks v2.2.1 (Cufflinks, RRID:SCR_014597). Then, all transcripts were merged using gffcompare^55^ to generate a transcriptome assembly. Protein coding genes were identified using TransDecoder (TransDecoder, RRID:SCR_017647). All scripts were written in Snakemake v5.26.1 (Snakemake, RRID:SCR_003475).

### Annotation of protein coding genes

To annotate the *P. ovata* genome, we ran three rounds of the MAKER v2.31.11 (MAKER, RRID:SCR_005309)^35^ pipeline with a combination of identified transcripts using TransDecoder (TransDecoder, RRID:SCR_017647), protein sequences from Viridiplantae, UniProtKb database (https://www.uniprot.org/taxonomy/33090), and ab initio gene predictors (SNAP v2013_11_19^56^ and AUGUSTUS v3.2.3^57^). The BUSCO v5.4.3^36^ pipeline was used for AUGUSTUS^57^ training. BLAST v2.13.0 (BLASTP, RRID:SCR_001010) with parameters ‘-evalue 1e-6 -max_hsps 1 -max_target_seqs 1’ was used to search homologous genes against a local database created from the UniProtKB database (Viridiplantae). Protein coding sequences (CDS) were extracted using the script agat_sp_extract_sequences.pl from AGAT^58^ followed by gaas_fasta_statistics.pl from Genome Assembly Annotation Service (GAAS)^53^ to calculate the GC content. We also used EMBOSS infoseq (EMBOSS, RRID:SCR_008493) to calculate the GC content of each CDS.

### Comparative genomic analysis and identification of glycosyltransferase (GT) 61 genes

OrthoFinder v2.5.4 (OrthoFinder, RRID:SCR_017118)^37^ was used to perform comparative genomic analysis on *P. ovata* protein-coding genes and nine other species (Supplementary File 1: Table S15). The synteny relationship between *P. ovata* and *A. majus* (snapdragon) was identified using MCscan (jcvi v1.2.11)^39^. Orthogroups and orthologous of eighteen genes from the glycosyltransferase (GT) 61 family previously identified in different *P. ovata* tissues, including mucilage-producing tissues^43,45^ were searched. Seven genes from *PoGT61_1* to *PoGT61_7* (KC894060 to KC894066) were obtained from Jensen et al.^45^. Eleven genes, namely *PoGT61_1L*, *PoGT61_4L*, *PoGT61_8* to *PoGT61_11*, *PoGT61_11L*, *PoGT61_12*, *PoGT61_13*, *PoGT61_17*, and *PoXYLT* (KY440071 to KY440081, respectively) were identified from Phan et al.^43^. EMBOSS Transeq (EMBOSS, RRID:SCR_008493) was used to translate nucleic acid sequence. Multiple sequence alignments using MUSCLE v3.8.1551 (MUSCLE, RRID:SCR_011812) were performed on GT61 protein sequences identified from OrthoFinder’s results. A phylogenetic tree was built from the aligned sequences using FastTree v2.1.10 (FastTree, RRID:SCR_015501) and visualised using FigTree v1.4.4 (FigTree, RRID:SCR_008515).

### Annotation of non-coding genes

Three non-coding RNA databases and three bioinformatics tools were used to search and annotate non-coding RNA using genomic and transcript sequences. A local database was built from RNAcentral^59^, a plant non-coding RNA database PNRD^60^, and CANTATAdb v2.0^61^ and sequence homologies identified using BLASTN (BLASTN, RRID:SCR_001598). We also used tools RNAMMER (RNAmmer, RRID:SCR_017075) for ribosomal RNA (rRNA), tRNAscan-SE v2.0.7 (tRNAscan-SE, RRID:SCR_010835) for transfer RNA (tRNA), and FEELnc^62^ for long non-coding RNA (lncRNA) detection.

## Supplementary Information

Supplementary Information 1.

Supplementary Information 2.

Supplementary Information 3.

Supplementary Information 4.

Supplementary Information 5.

Supplementary Information 6.

Supplementary Information 7.

Supplementary Information 8.

Supplementary Information 9.

Supplementary Information 10.

Supplementary Information 11.

Supplementary Information 12.

Supplementary Information 13.

Supplementary Information 14.

Supplementary Information 15.

Supplementary Information 16.

Supplementary Information 17.

## Data Availability

The datasets generated during this study were deposited in the NCBI SRA (Sequence Read Archive) database under the BioProject ID: PRJNA732452. The genome sequence data (PacBio and Hi-C) are available under accession numbers SRR14643405 and SRR14643406. Transcriptome data are available under accession numbers SRR14643399-SRR14643404 and SRR14643407-SRR14643436. Metadata and permanent links of previously published datasets analysed during the current study are listed in Supplementary File 1: Table S1. This Whole Genome Shotgun project has been deposited at DDBJ/ENA/GenBank under the accession JAHHQI000000000. The version described in this paper is version JAHHQI010000000. Annotation files for protein-coding genes, non-coding genes, and repeat and sequence files for transcripts and proteins can be found in Supplementary Files 13 to 17, respectively. Source code is available at GitHub https://github.com/herlianal12, and databases and software used for the analyses are included within the article (see also Supplementary File 1: Table S15 and Table S16).

## Abbreviations

AED: Annotation Edit Distance
AGRF: Australian Genome Research Facility Ltd
BLAST: Basic Local Alignment Search Tool
bp: Base pairs
BUSCO: Benchmarking Universal Single-Copy Orthologs
BWA: Burrows–Wheeler Aligner
CDS: Coding sequence
CLR: Continuous long read
CRL: Custom repeat library
DPA: Days post anthesis
FISH: Fluorescence in situ hybridization
Hi-C: High-throughput chromosome conformation capture
kb: Kilobase pairs
Gb: Gigabase pairs
LINE: Long interspersed nuclear element
lncRNA: Long non-coding RNA
LAI: LTR Assembly Index
LTR: Long terminal repeat
Mb: Megabase pairs
NCBI: National Center for Biotechnology Information
ncRNA: Non-coding RNA
NIB: Nuclei Isolation Buffer
NUMT: Nuclear mitochondrial
NUPT: Nuclear plastid
PacBio: Pacific Biosciences
RNA-seq: RNA sequencing
rRNA: Ribosomal RNA
SMRT: Single-molecule real-time
SRA: Sequence Read Archive
TE: Transposable element
TRF: Tandem Repeats Finder
tRNA: Transfer RNA
UTR: Untranslated region
