# Supplementary material for: A chromosome-level genome assembly of Plantago ovata
Source: Sci Rep. 2023 Jan 27;13:1528. doi: 10.1038/s41598-022-25078-5 (PMC9883528; doi:10.1038/s41598-022-25078-5)
Supplement: Supplementary file 2 — Supplementary Information 2. [file 41598_2022_25078_MOESM2_ESM.docx]

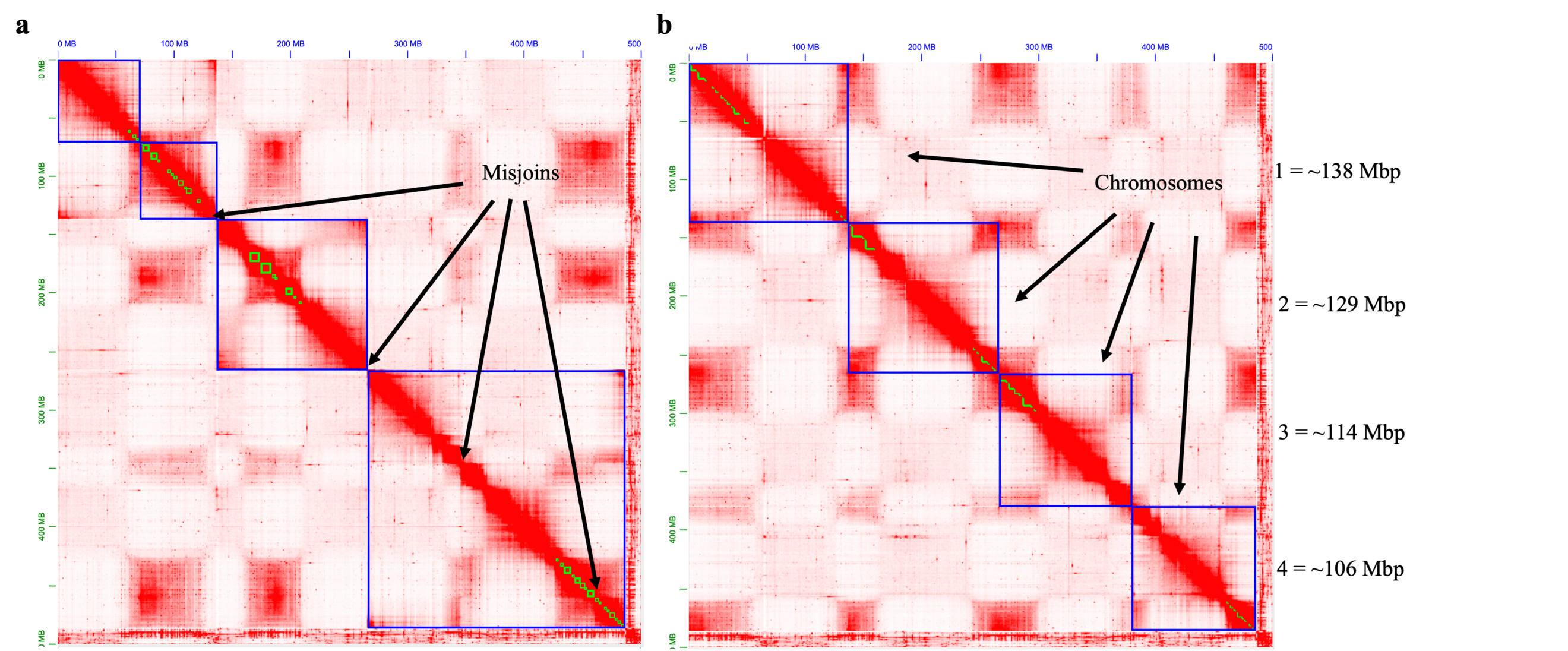


**Supplementary File 2:** Hi-C interaction heat map generated by 3D-DNA pipeline, visualised, and corrected by JBAT (Juicebox Assembly Tools). **a**. Before curation, we found misjoins in the assembly. **b.** After curation, we obtained four superscaffolds or chromosomes. Green boxes represent scaffolds while blue boxes refer to chromosomes.
