# Supplementary material for: A chromosome-level genome assembly of Plantago ovata
Source: Sci Rep. 2023 Jan 27;13:1528. doi: 10.1038/s41598-022-25078-5 (PMC9883528; doi:10.1038/s41598-022-25078-5)
Supplement: Supplementary file 3 — Supplementary Information 3. [file 41598_2022_25078_MOESM3_ESM.docx]

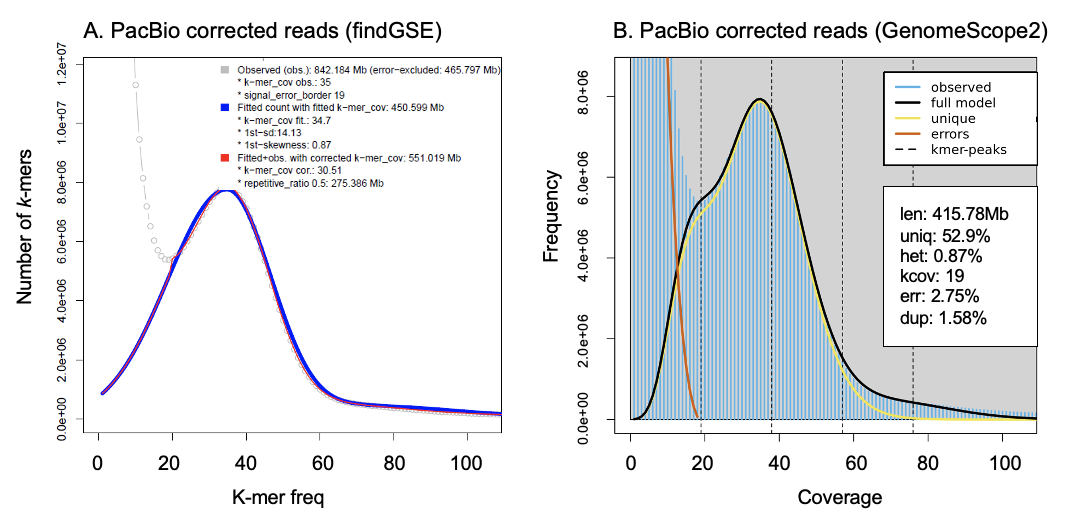


**Supplementary File 3:** Estimation of *P. ovata* genome size by analysing *k*-mers using two different methods.
