# Supplementary figures and images for: A chromosome-level genome assembly of Plantago ovata
Source: Sci Rep. 2023 Jan 27;13:1528. doi: 10.1038/s41598-022-25078-5 (PMC9883528; doi:10.1038/s41598-022-25078-5)

**
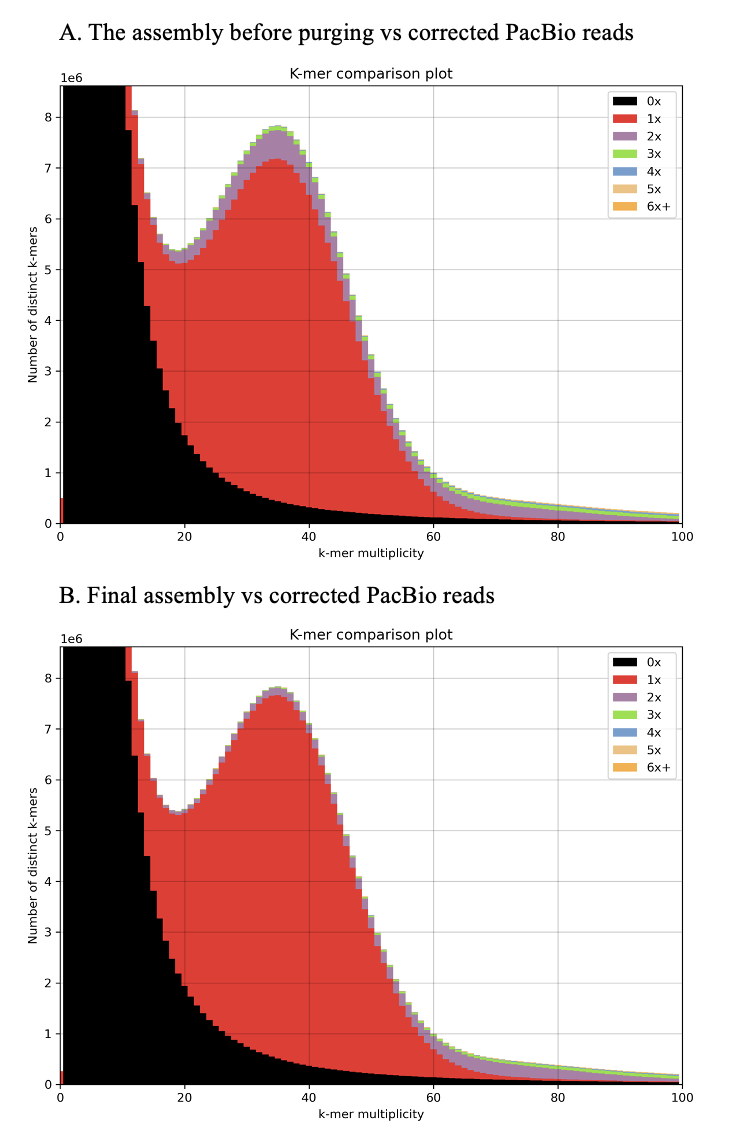
**

**Supplementary File 4:** Genome quality assessment using KAT comp.

Supplement: Supplementary file 4 — Supplementary Information 4. [file 41598_2022_25078_MOESM4_ESM.docx]

**Supplementary File 6:** Three locations of nuclear mitochondrial DNA (NUMT).


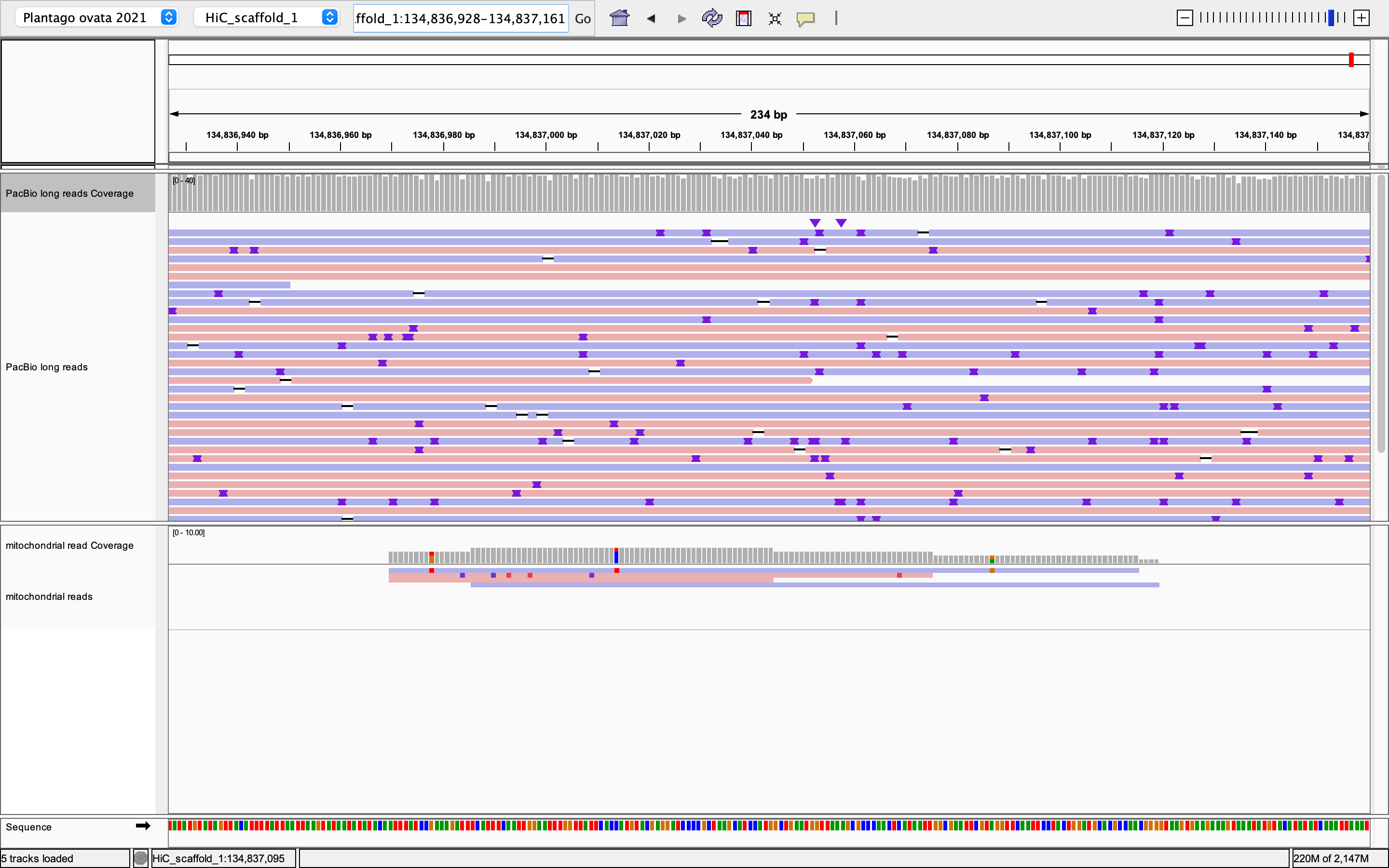


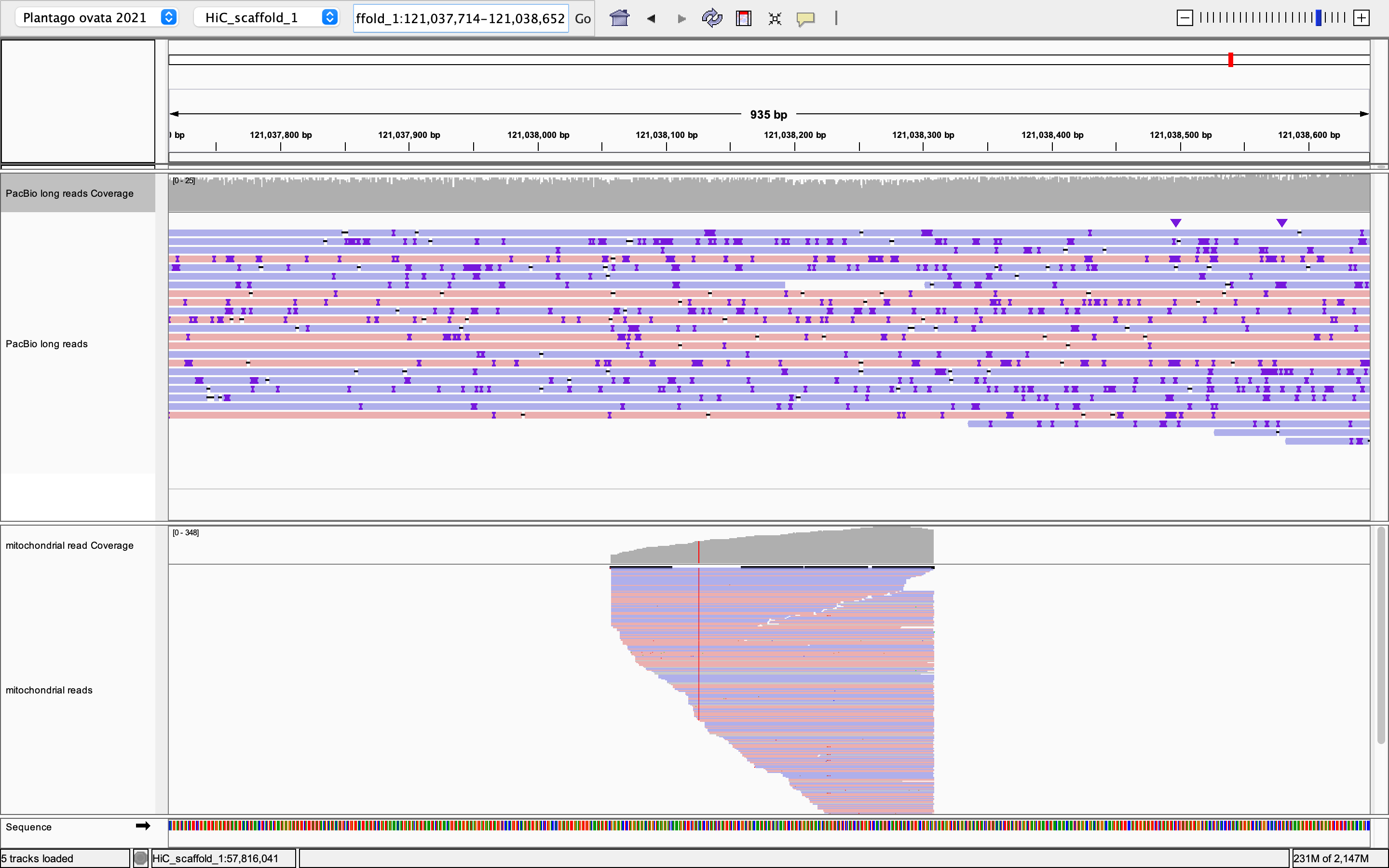


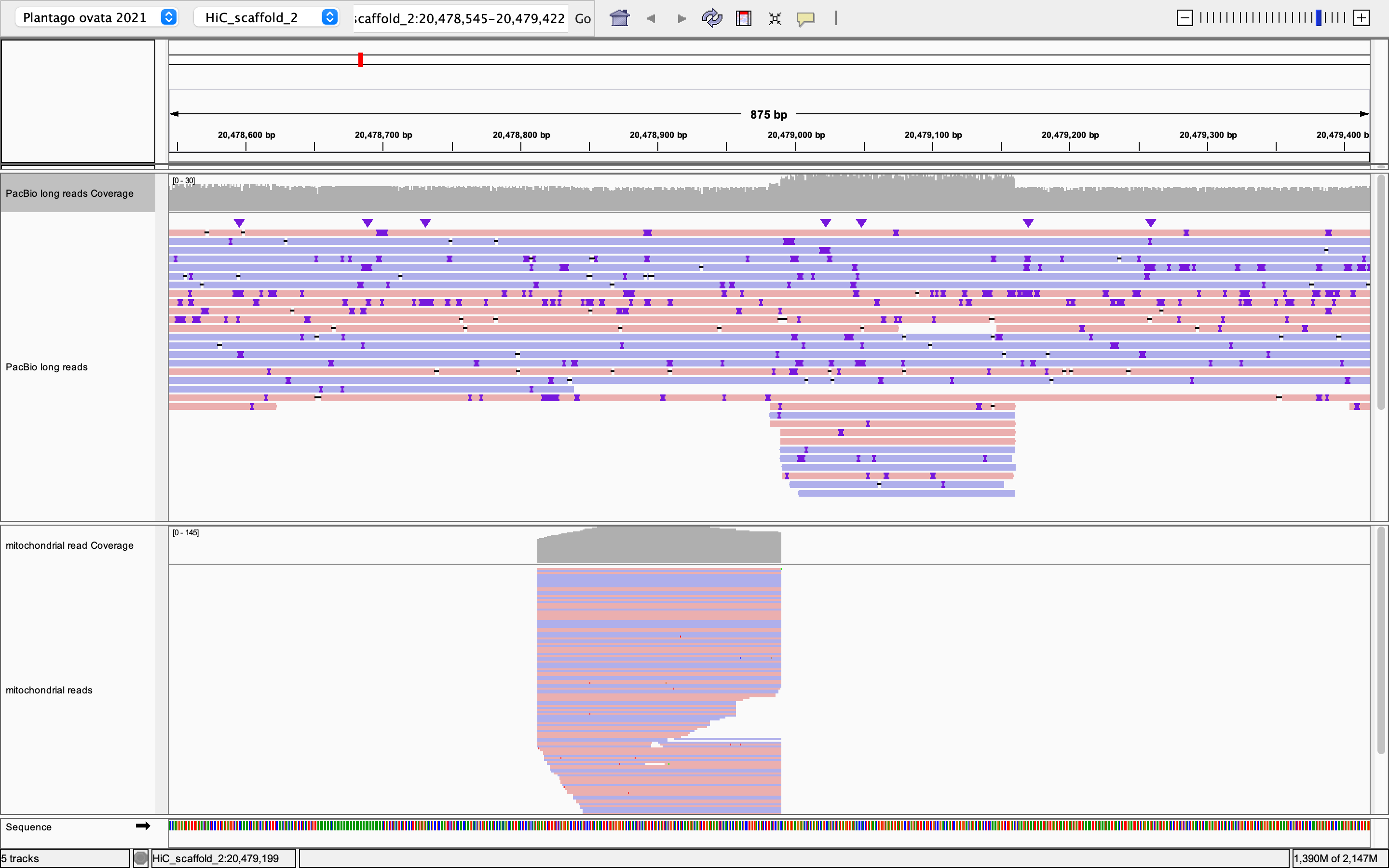

Supplement: Supplementary file 6 — Supplementary Information 6. [file 41598_2022_25078_MOESM6_ESM.docx]

**
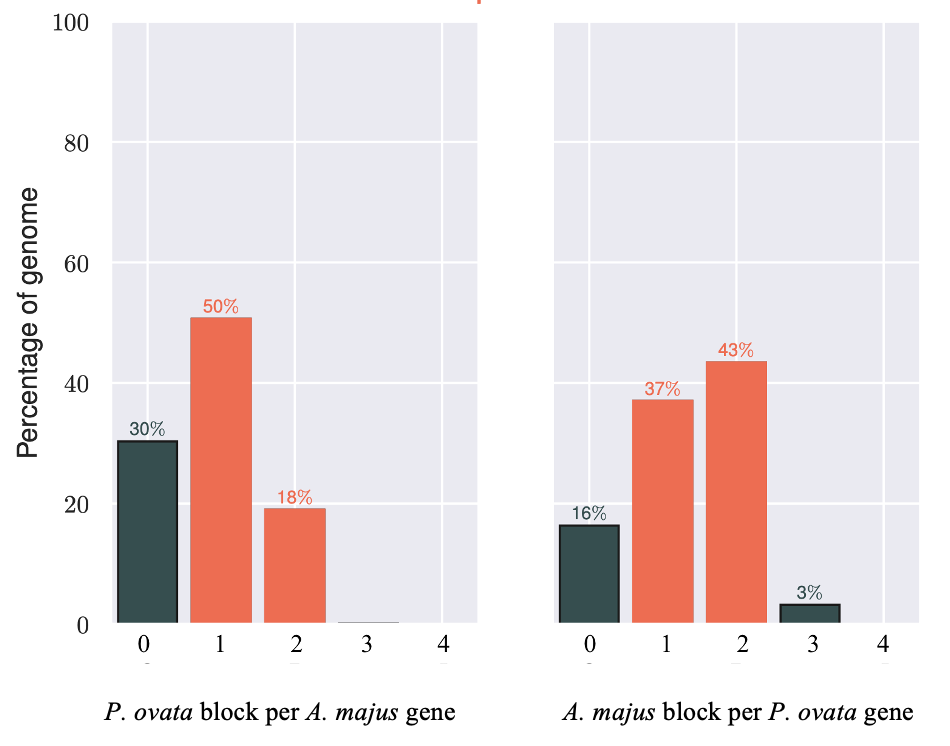
**

**Supplementary File 10:** Syntenic depths between *P. ovata* and *A. majus*

Supplement: Supplementary file 10 — Supplementary Information 10. [file 41598_2022_25078_MOESM10_ESM.docx]
