# Supplementary material for: A chromosome-level genome assembly of Plantago ovata
Source: Sci Rep. 2023 Jan 27;13:1528. doi: 10.1038/s41598-022-25078-5 (PMC9883528; doi:10.1038/s41598-022-25078-5)
Supplement: Supplementary file 7 — Supplementary Information 7. [file 41598_2022_25078_MOESM7_ESM.docx]

**Supplementary File 7:** Variant telomeric repeats in the *P. ovata* genome.

>Chromosome 1 [1-377] 377bp

5’AACCCCAAACCCTGAACCCGGAACCCTGAACCCTGAACCCTAAACCCTCAACCCCTCAACCCGGAACCCTCAACCCCTCACCCGGAACCCCTCAACCCGAACCGGAACCCGGAACCCGGAACCCGGAACCGGAACCCGGAACCTGAACCCTGAACCCTAAACCCTCAACCCCTCAACCGGAACCCTCAACCCGAACCCGGAACCCGGAACCCCTCAACCCGAACCCGGAACCCGGAACCCGGAACACGGAACCCGGAACCCGGAACCCCGAACCCCGAAACCCCGAAACCGAAAACCCCGAACACCTGAACCCCGAAACCCCGAAACCCGACCCCGAACCCGAACCCCGAAACCCTAAACCCTAAACCCTAAACCCT3’

>Chromosome 1 [137725100-137725283] 184bp

5’TTAGGGTTTGGGGTTGGGGTTGGGGGTTGGGGTTTTGGTTTGGGGTTTTAGGGTTTAGGGTTTAGGGGTTAGGGTTTAGGGTTTAGGGTTTGGGGTTTGGGGTTAGGGTTTAGGGTTTTAGGGTTTAGGGTTAGGGTTGGGGTTTGGGGTTTGGGGTTTGGGGTTTTGGGGTTGGGGTTTTGGG3’

>Chromosome 2 [4-362] 359bp

5’AAACCCTAAACCCTAAACCCTATAACCCTAAACCCTAAACCCTAAACCCAAAACCCTATAACCCAAAACCCTATAACCCAAAACCCTATAACCCAAAACCCTATAACCCAAAACCCCAAACCCAAAAACCCTATAACCCTAAACCCTATAACCCCAAACCCTATAACCCCAAACCCTATAACCCAAAACCCTATAACCCAAACCCTATAACCCAAAACCCTAAACCCTAAACCCTAAACCCCAAACCCAAAACCCTATAACCCCAAACCCTAAAACCCCAAACCCTATAACCCCAAACCCTATAACCCAAAACCCTATAACCCAAACCCTATAACCCTACACCCCAAACCCTAAACCCT3’

>Chromosome 2 [128866084-128866842] 759bp

5’TTTAGGTTTAGGGTTTAGGGTTTAGGGTTTAGGGTTTAGGGTTTAGGGTTTAGGGTTTAGGGTTTAGGGTTTAGGGTTTAGGGTTTAGGGTTTAGGGTTTAGGGTTTAGGGTTTAGGGTTTAGGGTTTAGGGTTTAGGGTTTAGGGTTTAGGGTTTAGGGTTTAGGGTTTAGGGTTTAGGGTTTAGGGTTTAGGGTTTAGGGTTTAGGGTTTAGGGTTTAGGGTTTAGGGTTTAGGGTTTAGGGTTTAGGGTTTAGGGTTTAGGGTTTAGGGTTTAGGGTTTAGGGTTTAGGGTTTAGGGTTTAGGGGTTTCGGTTTCGGGTTTAGGTTTCGGGTTTAGGGTTTAGGGTTCGGGTTCGGGTTTAGGGTTTCGGGTTAGGGTTTAGGTTAGGGTTTAGGGTAGGGTTAGGGGTTCGGGTTTAGGTTTAGGGTTGGGTTTTCGGTTGGGGTTTAGGGTTTAGGGTTTTAGGTTTAGGGTTGGGGTTTAGGTTTAGGGTTTTCGGTTTAGGGTTTAGGGTTTAGGGTTTAGGGTTTAGGGTTTTCGGTTTAGGGTTTAGGGTTTCGGGTTTATGGTTTAGGGTTTCGGGTTTCGGTTTCGGGTTTCGGGTTTCGGGTTTTCGGGTTTCGGGTTAGGGTTTACGGGTTTCGGGTTAGGGTTTCGGGTTATAGGTTTCGGGTTTCGGGTTTCGGTTCGGTTTAGGGTTTCCGGGTTTCGGTTTTCGGGTTTCGGGTTTGGGTTTCGGGTTTCGGGGTTTCGGGG3’

>Chromosome 2 [128867197-128867510] 313bp

5’AAACCCTACACCCTAAACCCACTAAACCTAAACCCTAAACCCAAACCCTAACCCCTAAACCCTAAACCCGAACCCTAAACCCTAAACCTAAACCCTAACCCCTCCCCCCTAACCCCTACCCTAAACCCTAAACCCTAAACCCTCAACCCTAAACCCTAAACCCTAAACGCCTAAACCCCTAAACCCTAAACCCTAACCCTAAACCCTAAACCCTAAACCCTAACCCCTAACCCCTAAACCCTAAAAACCCTAAACCCTCAAACCCTAAACCCTAAACCCTAAACCCTCAAACCCTAAACCCGAAACCCTAACCC3’

>Chromosome 3 [114444289-114444890] 602bp

5’TTTAGGGTTTCGGGGTTTGGGGTTCAGGGGTTTGGGGTTTTCGGGTTTTCGGGTTCGGGGTTGAGGGTTTAGGGTTTAGGGGTTCTGGGTTTGGGGTTCAGGGTTTAGGGTTTAGGGTTTAGGGTTTAGGGTTTAGGGTTCAGGGTTCAGGGTTCAGGGTTCAGGGTTCAGGGTTCAGGTTCAGGGTTCAGGGTTCAGGGTTCAGGGTTGAGGGTTGAGGGTTCAGGGTTCAGGGTTCAGGGTTCAGGGTTCAGGGTTCAGGTTCAGGGTTTAGGGTTTAGGGTTTAGGGTTAGGGTTTAGGGTTTAGGGTTTAGGGTTTAGGGTTTAGGGTTTAGGGTTTAGGGTTTAGGGTTTAGGGTTTAGGGTTTAGGGTTTAGGGTTTAGGGTTTAGGGTTTAGGGTTTAGGGTTTAGGGTTTAGGGTTTAGGGTTAGGGTTTAGGGTTTAGGGTTTAGGGTTTAGGGTTTAGGGTTTAGGGTTTAGGGGTTAAGGGTTTAGGTTAGGGTTTAGGGTTTGGGTTTAGGGTTTTAGGGGTTAGGGTTTAGGGTTTAGGGTTTAGGTTAGGGTTTAGGGTTTAGGGTTTAGGTTTAGGGTTTAGGGTTT3’

>Chromosome 4 [4-518] 515bp

5’AAACCCTAAACCCTAAACCCTAAACCCTAACCCTAAACCCTAAACCCTAAACCCTAAACCCTAAACCCTAAACCCTAAACCCTAAACCCTAAACCCTAAACCCTAAACCCTAAACCCTAAACCCTAAACCCTAAACCCTAAACCCTAAACCCTAAACCCTAAACCCTAAACCCTAAACCCTAAACCTAAACCCTAAACCCTAAACCCTAAACCCTAAACCCTAAACCCTAAACCCTAAACCCTAAACCCTAAACCCTAAACCCTAAACCCTAAACCCTAAACCCTAAACCCTAAACCCTAAACCCTAAACCCTAAACCCTAAACCCTAAACCCTAAACCCTAAACCCTAAACCCTAAACCCTAAACCCTAAACCCTAAACCCTAAACCCTAAACCCTAAACCCTAAACCCTAAACCCTAAACCCTAAACCCTAAACCCTAAACCCTAAACCCTAAACCCTAAACCCTAAACCCTAAACCCTAAACCCTAACCCTAAACCCTAAACCCTAAACCCT3’

>Chromosome 4 [106345914-106,346,329] 416bp

5’TTTAGGGTTTGGGGTTTTAGGGTTTTGGGGTTTTGGGGTTTTGGGGTTTTGGGTTTAGGGTTTGGGTTTTGGGGTTTTGGGTTTCGGGGTTTTAGGGTTTCGGGGTTTTAGGGTTTCGGGGTTTGGGTTTGGGGTTTCGGGTTTGGGGTTTCGGGGTTTGGGTTTGGGTTTTGGGGTTCTGGGTTTCGGGGTTTTAGGGTTTCGGGGTTTTAGGGTTTCGGGGTTTGGGTTTGGGTTTGGGGTTTAGGGTTTGGGGTTTCGGGGTTTGGGTTTGGGGTTTAGGGTTTTGGGGTTCTGGGTTTCGGGGTTTGGGTTTAGGGTTTGGGGTTTAGGGTTTAGGGTTTAGGGTTTAGGGTTTTAGGGTTTTAGGGTTTTAGGGTTTGAGGGTTTTAGGGTTTTAGGGTTTAGGGTTTTGG3’
