# Supplementary material for: A chromosome-level genome assembly of Plantago ovata
Source: Sci Rep. 2023 Jan 27;13:1528. doi: 10.1038/s41598-022-25078-5 (PMC9883528; doi:10.1038/s41598-022-25078-5)
Supplement: Supplementary file 12 — Supplementary Information 12. [file 41598_2022_25078_MOESM12_ESM.pdf]

Hi-C Library QC Report

Genome Scaffolding Sufficiency

Label	Library statistics	Expected values
Subjective Hi-C library judgment	**SUFFICIENT**	See Judgment
Same strand high-quality* (HQ) read pairs (RPs)	**24.89%**	> 1.5%
Informative RPs**	**27.67%**	> 5.0%

*High quality (HQ) read pairs have minimum mapping quality >= 20, maximum edit distance <= 5, and are not duplicates.
**Informative read pairs are read pairs which have MAPQ > 0, are not PCR duplicates, and map to different contigs or >10 Kbp apart.

Metrics Demonstrating Strong Proximity Signal

Label	Library statistics	Expected values
Fraction of HQ RPs >10KB apart (CTGs >10KB)*	**25.42%**	> 3.0%
Fraction of HQ RPs Intercontig on CTGs >10KB**	**33.37%**	> 2.5%
Clustering usable HQ reads per contig (CTGs >5KB)***	**33.29**	> 600.0

*The proportion of *read pairs that span at least 10kbp*, out of all read pairs that map (a) with high-quality, (b) to the same contig, (c) where that contig is at least 10kbp long.
**The proportion of *read pairs mapping to two different contigs each greater than 10kbp*, out of all read pairs that map with high-quality.
***The average number of usable high-quality read pairs per contig, for contigs greater than 5kbp. Read pairs are "usable" if they map (a) with high-quality, (b) to different contigs, (c) where each of those contigs are greater than 5kbp and (c) both mappings are high-quality.

See below for information on differences between Phase Genomics Hi-C libraries and traditional Hi-C libraries.

Noninformative Read Pair Breakdown

Label	Library statistics	Expected values
Noninformative RPs*	**50.44%**	<= 50.0%
Duplicate reads	**0.95%**	< 20.0%
Zero map distance read pairs	**2.12%**	<= 20.0%
Zero MAPQ reads	**33.88%**	<= 20.0%
Unmapped reads	**0.00%**	<= 10.0%

*Note that the sum of informative and noninformative read pairs is not 100% because read pairs with mapping distance between 1 and 10 Kbp are not classified as either informative or noninformative.
Because noninformative reads can belong to more than one category, these numbers may sum to a value larger than the overall noninformative read pair amount at the top of the report.

See below for information on differences between Phase Genomics Hi-C libraries and traditional Hi-C libraries.

Assembly Statistics

Label	Assembly statistics
BAM file	Povata-HiC_combined.bam
Assembly size	500,599,874
Contig (CTG) N50	260,246
CTGs	3,919
CTGs > 10KB	3,800
CTGs > 5KB	3,859

Extended Library Statistics

Label	Library statistics	Expected values
Total read pairs (RPs) analyzed	1,000,001	N/A
High quality (HQ) RPs	38.37%	N/A
RPs >10KB apart	10.23%	1-15%
RPs >10KB apart (CTGs >10KB)	26.03%	1-15%
Intercontig RPs	60.64%	10-60% (contigs) 1-20% (chromosomes)
Intercontig HQ RPs	33.55%	10-60% (contigs) 1-20% (chromosomes)
Same strand RPs	24.97%	2-50%
Split reads	25.37%	1-10% (PG libraries) 30%+ (other libraries)
Duplicate reads (extrapolated)*	9.67%	0-50%

*Extrapolated to 100,000,000 RPs. If extrapolation fails, it will be -1%.

Aligned mate distance histograms

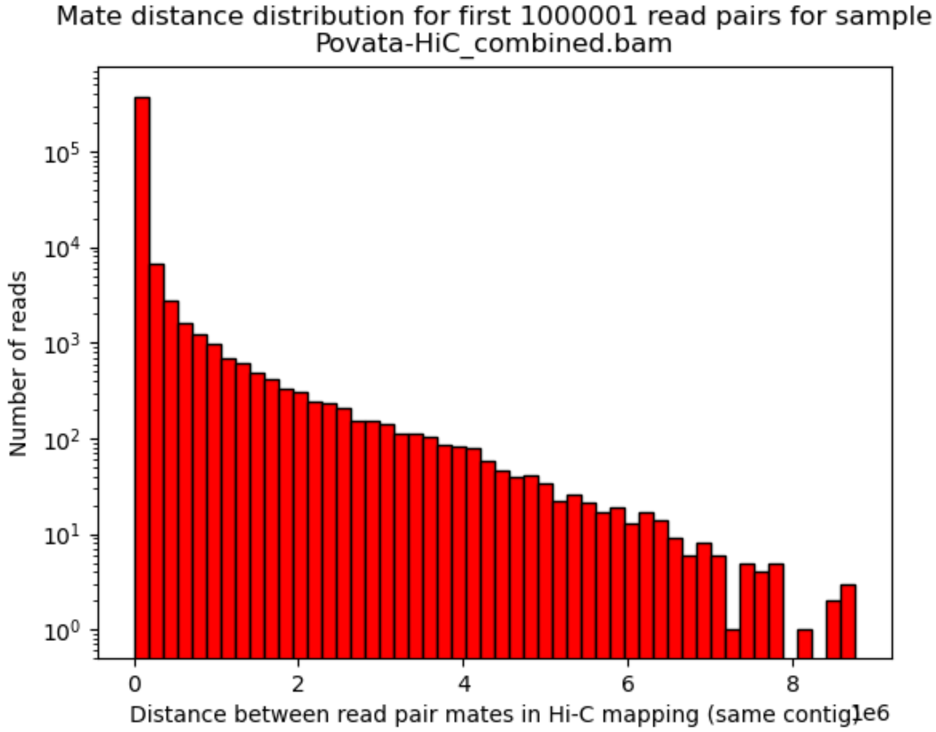

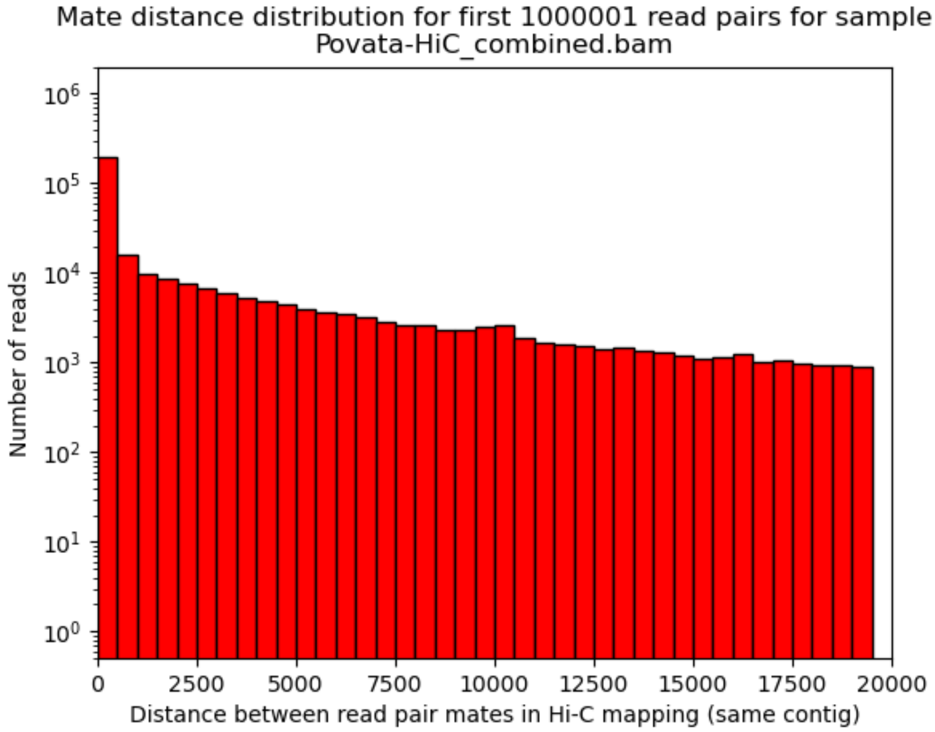

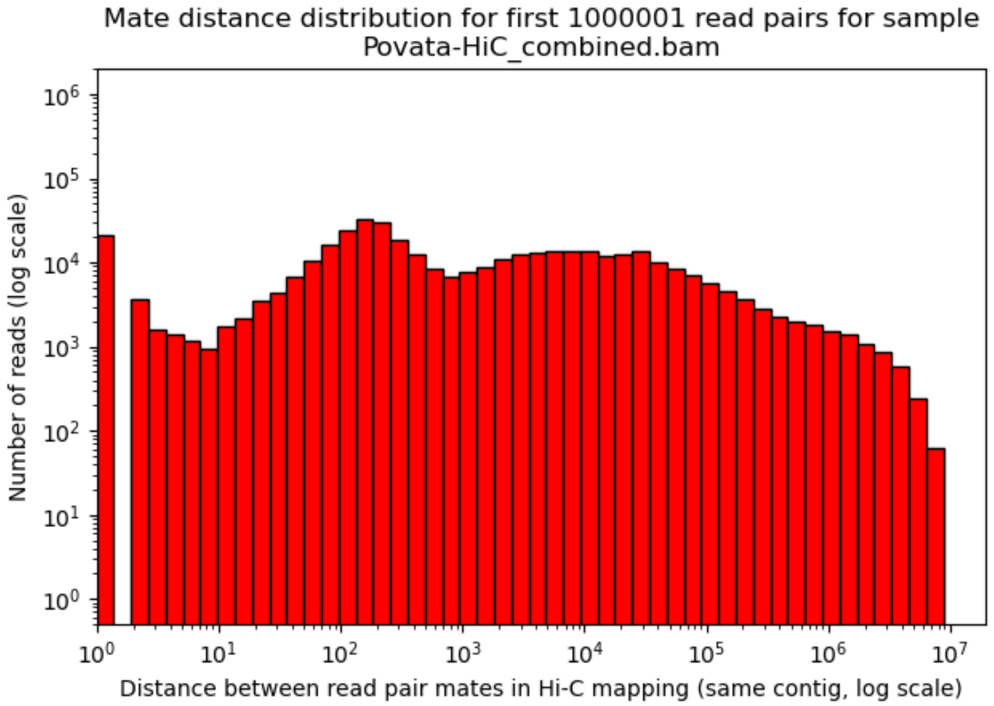
Mate distance distribution for first 1000001 read pairs for sample
Povata-HiC_combined.bam
Number of reads (log scale)
Distance between read pair mates in Hi-C mapping (same contig, log scale)

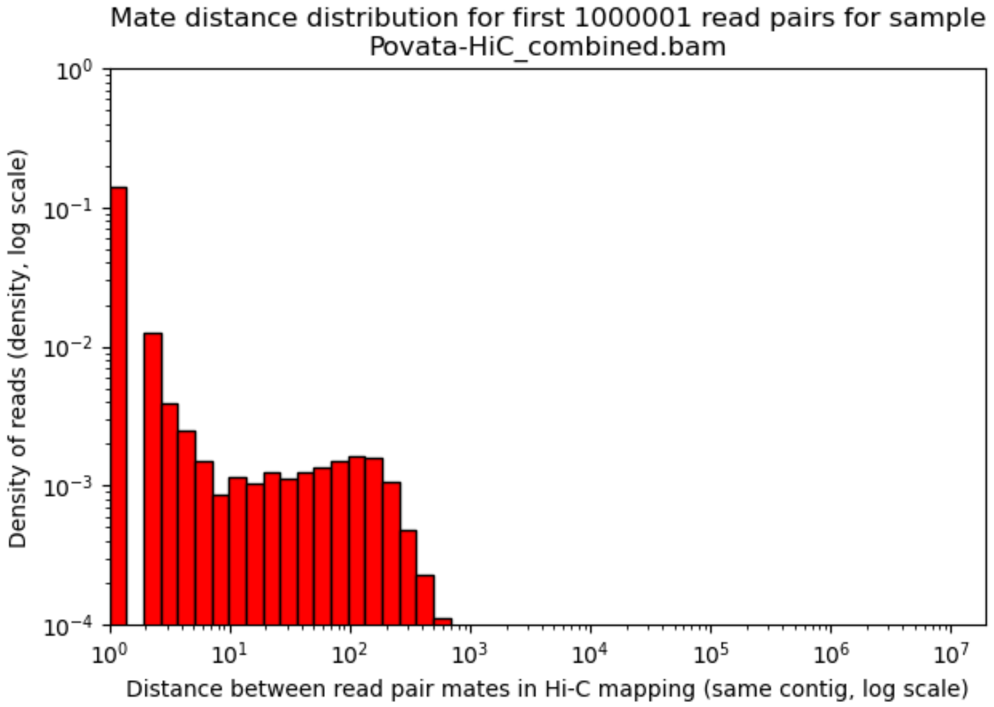
Mate distance distribution for first 1000001 read pairs for sample
Povata-HiC_combined.bam
Density of reads (density, log scale)
Distance between read pair mates in Hi-C mapping (same contig, log scale)

Duplicate read saturation curve

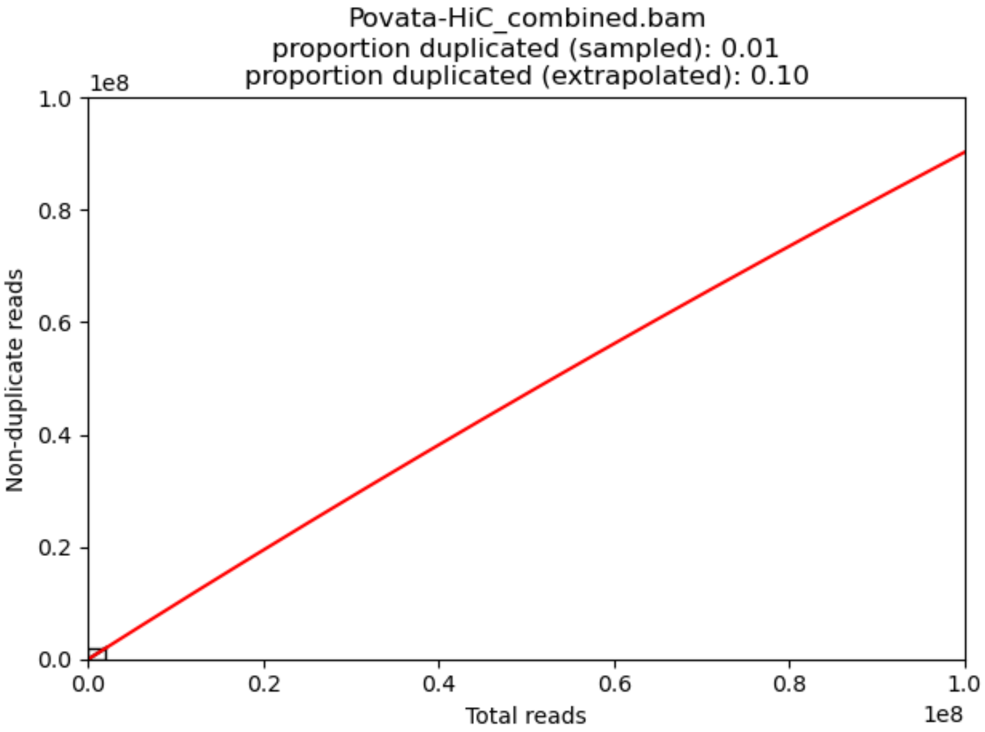

Alignment distance statistics and plots

We briefly describe some of the statistics we compute below to aid interpretation of this report.

Subjective Hi-C Library Judgment

While Hi-C data is nuanced and some analyses are more sensitive to data quality than others, a basic quality assessment can usually be made by examining the mapping characteristics of the Hi-C library. Based on our experience working with Hi-C data, we classify libraries into one of four QC categories:
- **Sufficient** means that from everything we can tell, the library looks to be in great shape. Proceed to full sequencing or analysis with confidence.
- **Mixed Results** means that the library is good in some ways, but not in others. Perhaps it has a good amount of long range data, but there are also an elevated number of read pairs with MAPQ 0. Usually, data generated from Mixed Results libraries works out just fine (a high MAPQ 0 number can be due to repetitiveness in the assembly or unpurged haplotigs, for example), but it is good to know there may have been a few hiccups in the library prep in case troubleshooting is needed down the line.
- **Low Signal** means that the library contains good Hi-C signal, but it's in lower percentage than usual. These libraries are generally good for generating useful Hi-C data, but you may need to sequence a little deeper than normal to get enough of it. You might consider size selecting the library to discard reads outside the 300-700bp range, as these are unlikely to be good Hi-C junctions. Alternatively, you might just want to prep a new library.
- **Insufficient** means that the library, or perhaps the library in combination with a low-contiguity or error-prone assembly, does not look useful. Sometimes size selection can rescue such libraries, but sometimes a new prep is the only way forward.

Contact us if your library is not sufficient and we will help you out.

Read pairs on same strand

This is the percentage of reads mapping to the same contig in the same orientation (FF or RR). For shotgun libraries, this should be ~1%, but for a pure Hi-C library, it could be as high as 50%. This is a primary metric of Hi-C library quality because it is minimally affected by assembly contiguity.

Read pairs > 10kbp apart

This is the percentage of read pairs which map to the same contig, with at least 10kbp separating them. More is always better, but because this number is affected by assembly contiguity, there is not a specific target threshold. Note that for some analyses, such as scaffolding or metagenomic deconvolution, read pairs that map to the same contig are not useful because they do not provide information that the assembly doesn't already contain. This statistic is more useful for these projects because it correlates with library prep success. These reads are useful for analyses like structural variant analysis or assembly misjoin detection, because they provide detailed structural information about existing assembled sequences.

Read pairs > 10kbp apart mapping to contigs >10kbp

This is the percentage of read pairs which map to the same contig, with at least 10kbp separating them, but only considering read pairs mapping to contigs that are at least 10kbp long. This attempts to corrects for assembly contiguity differences. More is always better, but typically at least 5% is desired. Note that for some analyses, such as scaffolding or metagenomic deconvolution, read pairs that map to the same contig are not useful because they do not provide information that the assembly doesn't already contain. This statistic is more useful for these projects because it correlates with library prep success. These reads are useful for analyses like structural variant analysis or assembly misjoin detection, because they provide detailed structural information about existing assembled sequences.

Read pairs mapping to different contigs or chromosomes

This is the percentage of read pairs which map to different contigs, which is particularly important. More is always better, but because this number is affected by assembly quality, there is not a specific target threshold, although at least 20% on *de novo* assembly projects is helpful. These reads are the primary source of information for Hi-C scaffolding or metagenomic deconvolution analyses. This statistic useful on most *de novo* projects because it also correlates with library prep success. These reads may be useful for analyses like structural variant analysis or assembly misjoin detection if those are performed on lower contiguity assemblies, because they provide detailed structural information about sequences which were not assembled together into contigs.

Split reads

Traditionally, split reads have been a favored measure of Hi-C library quality because they directly exhibit Hi-C junctions. Most traditional Hi-C library preparations produce many reads that sequence through junctions because their Hi-C junctions tend to occur randomly on the proximity ligated chimeric molecules. However, Phase Genomics libraries, whether produced in our laboratory or by means of our Plant, Animal,

Human, or Microbe Hi-C kits, will have a generally lower percentage of split reads. This is because we have optimized our Hi-C protocol to enrich for slightly longer fragments around Hi-C junctions, such that each read is less likely to read through a junction even when a junction is present.

This innovation improves mappability and increases the amount of useful data, and reduces the utility of split read measurements to assess library quality. We therefore rely more heavily on metrics that directly relate to the usefulness of Hi-C reads for proximity analysis, such as the percentage of read pairs that map to the same strand or to different contigs.

Duplicate reads

IMPORTANT NOTE: THE DUPLICATE FLAG IS NOT SET BY DEFAULT IN A BAM FILE. YOU NEED TO EXPLICITLY SET IT BY E.G. RUNNING SAMBLASTER OR PICARD MARKDUPLICATES ON YOUR BAM FILE. IF THE PERCENT OF DUPLICATES IS EXACTLY ZERO, IT PROBABLY MEANS THAT THE FLAG HAS NOT BEEN SET.

Sequencing libraries frequently contain duplicate reads due to PCR or optical issues. These are generally considered to be non-informative because they are chemical artifacts rather than biological signal, and are thus typically excluded from further analysis. Higher percentages of duplicate reads are also correlated with low library complexity and poor library performance, making the percentage of duplicate reads a useful quality control measure.

Also, because we often recommend sequencing a few million read pairs for QC prior to a full sequencing run, it is helpful to project the amount of duplicate reads a full run might generate. To do this, we attempt to fit a curve to the rate at which duplicates are observed in a library, and then project the percentage of duplicates that would be expected in a deeper sequencing run. This projection is a reasonably useful heuristic, but it is not a guarantee that the true duplicate rate will be near a specific value. QC sequencing data with a very low number of reads or which mapped well at a very low rate can distort this calculation. We attempt to identify low confidence calculations and report that we could not extrapolate the expected duplicate frequency when it is possible to do so.

We use the function $f(x) = V * x / (x + K)$ to fit V and K, then extrapolate to the target number of reads (100,000,000 RPs).

Unmapped reads

A high percent of unmapped reads may indicate sequence is missing from the reference, the reads are mapped to the wrong reference, or the sample is contaminated.

REPORT VERSION: 0+untagged.183.g1e132fb

Hi-C Library QC Report

Genome Scaffolding Sufficiency

Label	Library statistics	Expected values
Subjective Hi-C library judgment	**SUFFICIENT**	See Judgment
Same strand high-quality* (HQ) read pairs (RPs)	**25.57%**	> 1.5%
Informative RPs**	**50.93%**	> 5.0%

*High quality (HQ) read pairs have minimum mapping quality >= 20, maximum edit distance <= 5, and are not duplicates.
**Informative read pairs are read pairs which have MAPQ > 0, are not PCR duplicates, and map to different contigs or >10 Kbp apart.

Metrics Demonstrating Strong Proximity Signal

Label	Library statistics	Expected values
Fraction of HQ RPs >10KB apart (CTGs >10KB)*	**26.01%**	> 3.0%
Fraction of HQ RPs Intercontig on CTGs >10KB**	**33.51%**	> 2.5%
Clustering usable HQ reads per contig (CTGs >5KB)***	**87.09**	> 600.0

*The proportion of *read pairs that span at least 10kbp*, out of all read pairs that map (a) with high-quality, (b) to the same contig, (c) where that contig is at least 10kbp long.
**The proportion of *read pairs mapping to two different contigs each greater than 10kbp*, out of all read pairs that map with high-quality.
***The average number of usable high-quality read pairs per contig, for contigs greater than 5kbp. Read pairs are "usable" if they map (a) with high-quality, (b) to different contigs, (c) where each of those contigs are greater than 5kbp and (c) both mappings are high-quality.

See below for information on differences between Phase Genomics Hi-C libraries and traditional Hi-C libraries.

Noninformative Read Pair Breakdown

Label	Library statistics	Expected values
Noninformative RPs*	**3.73%**	<= 50.0%
Duplicate reads	**0.00%**	< 20.0%
Zero map distance read pairs	**3.73%**	<= 20.0%
Zero MAPQ reads	**0.00%**	<= 20.0%
Unmapped reads	**0.00%**	<= 10.0%

*Note that the sum of informative and noninformative read pairs is not 100% because read pairs with mapping distance between 1 and 10 Kbp are not classified as either informative or noninformative.
Because noninformative reads can belong to more than one category, these numbers may sum to a value larger than the overall noninformative read pair amount at the top of the report.

See below for information on differences between Phase Genomics Hi-C libraries and traditional Hi-C libraries.

Assembly Statistics

Label	Assembly statistics
BAM file	Povata-HiC_filter.bam
Assembly size	500,599,874
Contig (CTG) N50	260,246
CTGs	3,919
CTGs > 10KB	3,800
CTGs > 5KB	3,859

Extended Library Statistics

Label	Library statistics	Expected values
Total read pairs (RPs) analyzed	1,000,001	N/A
High quality (HQ) RPs	100.00%	N/A
RPs >10KB apart	17.25%	1-15%
RPs >10KB apart (CTGs >10KB)	26.05%	1-15%
Intercontig RPs	33.67%	10-60% (contigs) 1-20% (chromosomes)
Intercontig HQ RPs	33.67%	10-60% (contigs) 1-20% (chromosomes)
Same strand RPs	25.57%	2-50%
Split reads	22.95%	1-10% (PG libraries) 30%+ (other libraries)
Duplicate reads (extrapolated)*	0.00%	0-50%

*Extrapolated to 100,000,000 RPs. If extrapolation fails, it will be -1%.

Aligned mate distance histograms

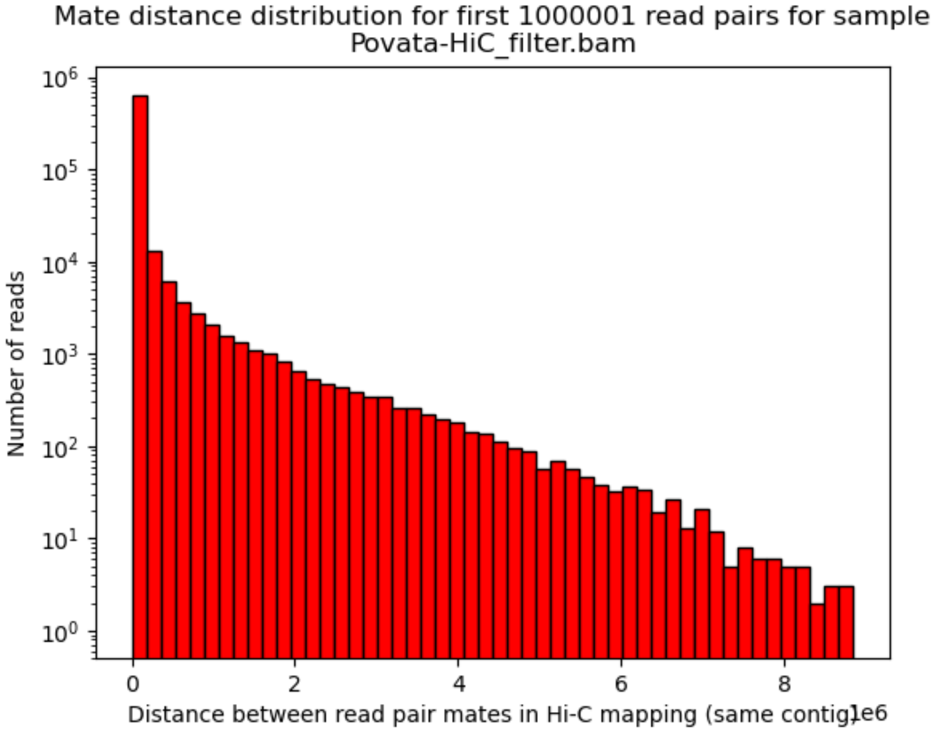

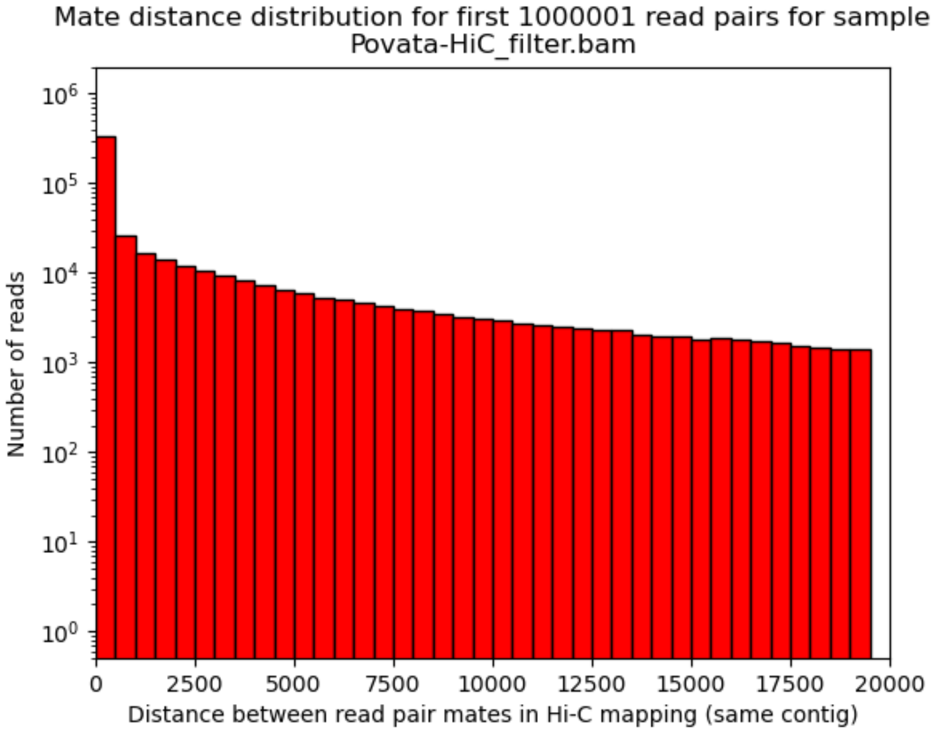

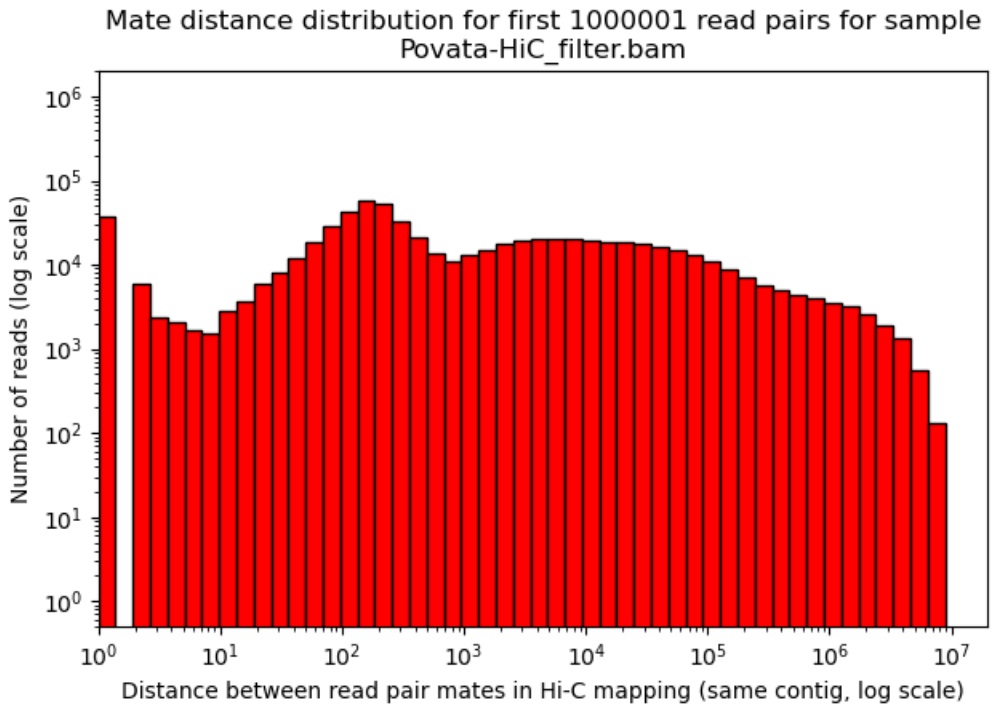
Mate distance distribution for first 1000001 read pairs for sample
Povata-HiC_filter.bam
Number of reads (log scale)
Distance between read pair mates in Hi-C mapping (same contig, log scale)

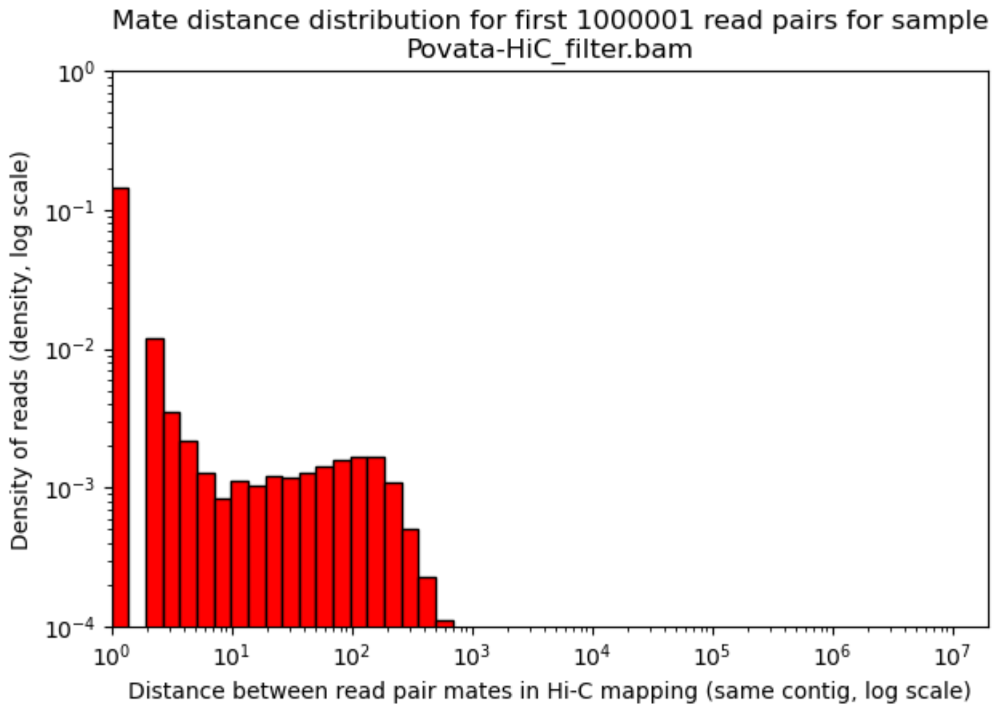
Mate distance distribution for first 1000001 read pairs for sample
Povata-HiC_filter.bam
Density of reads (density, log scale)
Distance between read pair mates in Hi-C mapping (same contig, log scale)

Duplicate read saturation curve

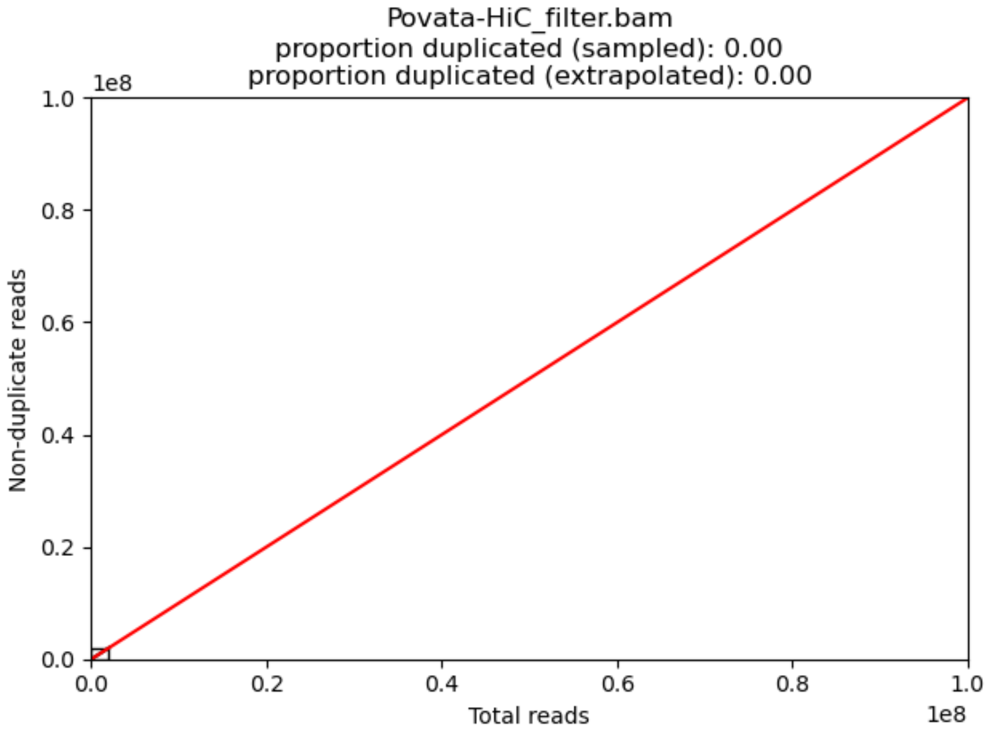

Alignment distance statistics and plots

We briefly describe some of the statistics we compute below to aid interpretation of this report.

Subjective Hi-C Library Judgment

While Hi-C data is nuanced and some analyses are more sensitive to data quality than others, a basic quality assessment can usually be made by examining the mapping characteristics of the Hi-C library. Based on our experience working with Hi-C data, we classify libraries into one of four QC categories:
- **Sufficient** means that from everything we can tell, the library looks to be in great shape. Proceed to full sequencing or analysis with confidence.
- **Mixed Results** means that the library is good in some ways, but not in others. Perhaps it has a good amount of long range data, but there are also an elevated number of read pairs with MAPQ 0. Usually, data generated from Mixed Results libraries works out just fine (a high MAPQ 0 number can be due to repetitiveness in the assembly or unpurged haplotigs, for example), but it is good to know there may have been a few hiccups in the library prep in case troubleshooting is needed down the line.
- **Low Signal** means that the library contains good Hi-C signal, but it's in lower percentage than usual. These libraries are generally good for generating useful Hi-C data, but you may need to sequence a little deeper than normal to get enough of it. You might consider size selecting the library to discard reads outside the 300-700bp range, as these are unlikely to be good Hi-C junctions. Alternatively, you might just want to prep a new library.
- **Insufficient** means that the library, or perhaps the library in combination with a low-contiguity or error-prone assembly, does not look useful. Sometimes size selection can rescue such libraries, but sometimes a new prep is the only way forward.

Contact us if your library is not sufficient and we will help you out.

Read pairs on same strand

This is the percentage of reads mapping to the same contig in the same orientation (FF or RR). For shotgun libraries, this should be ~1%, but for a pure Hi-C library, it could be as high as 50%. This is a primary metric of Hi-C library quality because it is minimally affected by assembly contiguity.

Read pairs > 10kbp apart

This is the percentage of read pairs which map to the same contig, with at least 10kbp separating them. More is always better, but because this number is affected by assembly contiguity, there is not a specific target threshold. Note that for some analyses, such as scaffolding or metagenomic deconvolution, read pairs that map to the same contig are not useful because they do not provide information that the assembly doesn't already contain. This statistic is more useful for these projects because it correlates with library prep success. These reads are useful for analyses like structural variant analysis or assembly misjoin detection, because they provide detailed structural information about existing assembled sequences.

Read pairs > 10kbp apart mapping to contigs >10kbp

This is the percentage of read pairs which map to the same contig, with at least 10kbp separating them, but only considering read pairs mapping to contigs that are at least 10kbp long. This attempts to corrects for assembly contiguity differences. More is always better, but typically at least 5% is desired. Note that for some analyses, such as scaffolding or metagenomic deconvolution, read pairs that map to the same contig are not useful because they do not provide information that the assembly doesn't already contain. This statistic is more useful for these projects because it correlates with library prep success. These reads are useful for analyses like structural variant analysis or assembly misjoin detection, because they provide detailed structural information about existing assembled sequences.

Read pairs mapping to different contigs or chromosomes

This is the percentage of read pairs which map to different contigs, which is particularly important. More is always better, but because this number is affected by assembly quality, there is not a specific target threshold, although at least 20% on *de novo* assembly projects is helpful. These reads are the primary source of information for Hi-C scaffolding or metagenomic deconvolution analyses. This statistic useful on most *de novo* projects because it also correlates with library prep success. These reads may be useful for analyses like structural variant analysis or assembly misjoin detection if those are performed on lower contiguity assemblies, because they provide detailed structural information about sequences which were not assembled together into contigs.

Split reads

Traditionally, split reads have been a favored measure of Hi-C library quality because they directly exhibit Hi-C junctions. Most traditional Hi-C library preparations produce many reads that sequence through junctions because their Hi-C junctions tend to occur randomly on the proximity ligated chimeric molecules. However, Phase Genomics libraries, whether produced in our laboratory or by means of our Plant, Animal,

Human, or Microbe Hi-C kits, will have a generally lower percentage of split reads. This is because we have optimized our Hi-C protocol to enrich for slightly longer fragments around Hi-C junctions, such that each read is less likely to read through a junction even when a junction is present.

This innovation improves mappability and increases the amount of useful data, and reduces the utility of split read measurements to assess library quality. We therefore rely more heavily on metrics that directly relate to the usefulness of Hi-C reads for proximity analysis, such as the percentage of read pairs that map to the same strand or to different contigs.

Duplicate reads

IMPORTANT NOTE: THE DUPLICATE FLAG IS NOT SET BY DEFAULT IN A BAM FILE. YOU NEED TO EXPLICITLY SET IT BY E.G. RUNNING SAMBLASTER OR PICARD MARKDUPLICATES ON YOUR BAM FILE. IF THE PERCENT OF DUPLICATES IS EXACTLY ZERO, IT PROBABLY MEANS THAT THE FLAG HAS NOT BEEN SET.

Sequencing libraries frequently contain duplicate reads due to PCR or optical issues. These are generally considered to be non-informative because they are chemical artifacts rather than biological signal, and are thus typically excluded from further analysis. Higher percentages of duplicate reads are also correlated with low library complexity and poor library performance, making the percentage of duplicate reads a useful quality control measure.

Also, because we often recommend sequencing a few million read pairs for QC prior to a full sequencing run, it is helpful to project the amount of duplicate reads a full run might generate. To do this, we attempt to fit a curve to the rate at which duplicates are observed in a library, and then project the percentage of duplicates that would be expected in a deeper sequencing run. This projection is a reasonably useful heuristic, but it is not a guarantee that the true duplicate rate will be near a specific value. QC sequencing data with a very low number of reads or which mapped well at a very low rate can distort this calculation. We attempt to identify low confidence calculations and report that we could not extrapolate the expected duplicate frequency when it is possible to do so.

We use the function $f(x) = V * x / (x + K)$ to fit V and K, then extrapolate to the target number of reads (100,000,000 RPs).

Unmapped reads

A high percent of unmapped reads may indicate sequence is missing from the reference, the reads are mapped to the wrong reference, or the sample is contaminated.

REPORT VERSION: 0+untagged.183.g1e132fb
